# Improved Charge Transport and Device Performance in p‐i‐n Carbon Electrode Perovskite Solar Cells Using SiC‐APTES Additive

**DOI:** 10.1002/smll.74393

**Published:** 2026-07-06

**Authors:** Mikhail Pylnev, Ryosuke Nishikubo, Tomoya Nakamura, Ryoji Shimomura, Atsushi Wakamiya, Akinori Saeki

**Affiliations:** ^1^ Department of Applied Chemistry Graduate School of Engineering The University of Osaka 2‐1 Yamadaoka Suita Osaka Japan; ^2^ Department of New Energy Science and Engineering School of Energy and Chemical Engineering Xiamen University Malaysia Sepang Selangor Malaysia; ^3^ Innovative Catalysis Science Division Institute For Open and Transdisciplinary Research Initiatives (ICS‐OTRI) The University of Osaka 1‐1 Yamadaoka Suita Osaka Japan; ^4^ Institute for Chemical Research Kyoto University Uji Kyoto Japan

**Keywords:** 3‐aminopropyltriethoxysilane (APTES), carbon paste electrode, lead iodide perovskite solar cells, p‐i‐n inverted device structure, silicon carbide

## Abstract

Carbon‐based electrodes offer a low‐cost and stable alternative to noble metals in lead halide perovskite solar cells (PSCs), yet their integration into high‐performing planar p‐i‐n architectures remains limited by poor interfacial contact and processing‐induced damage to thin electron transport layers (ETLs). Here, we introduce 3‐aminopropyltriethoxysilane (APTES)‐functionalized cubic SiC (SiC‐APTES) nanoparticles as a novel ETL‐like additive for carbon pastes (CP). Surface functionalization enables uniform dispersion, dramatically improves the rheology of CP, and promotes intimate contact with the ETL. The SiC‐APTES additive to CP boosts the power conversion efficiency from 14.75% to 18.43%, by reducing the series resistance and hysteresis. The resulting devices exhibit good stability under ambient operation, positioning the SiC‐APTES additive as an effective strategy for low‐cost p‐i‐n carbon‐electrode PSCs toward future scalability.

## Graphical abstract

Modification of carbon electrode by mixing silicon carbide nanoparticles functionalized with 3‐aminopropyltriethoxysilane improves the charge transport and device performance of p‐i‐n perovskite solar cells over 18% along with the decreased hysteresis and extended stability. We discuss the additive effect from rheological and electric measurements.

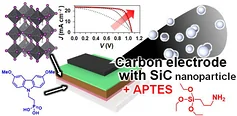

## 1 Introduction

Perovskite solar cells (PSCs) have reached power conversion efficiencies (PCEs) exceeding 21% in large‐area modules, making them one of the most promising next‐generation photovoltaic technologies [1]. However, the reliance on organic hole transport materials (HTMs) and noble‐metal electrodes (Au, Ag) remains a critical barrier to long‐term stability and low‐cost manufacturing [2]. This is because noble‐ metal deposition steps are among the major contributors to fabrication cost and scalability limitations [2]. Moreover, noble metals can diffuse across the hole transport layer (HTL) and lead to a permanent cell degradation [3, 4]. In particular, Ag electrodes can react with out‐diffusing halides and form silver iodide, which further impacts the overall PSC performance [5].

Carbon electrode‐based perovskite solar cells (C‐PSCs) have attracted much attention due to low‐cost and large‐scale fabrication [2, 6, 7]. Carbon pastes consisting of graphite flakes and carbon black have a work function of ∼5.0 eV and thus can be used for hole collection in conventional n‐i‐p PSCs [8]. Also, carbon electrodes are chemically inert, enabling excellent operational stability [9, 10]. C‐PSCs were the first reported over a decade ago [11] and have recently reached 21%–23% PCEs for a small‐size conventional n‐i‐p structure [12, 13].

The inverted (p‐i‐n) architecture has recently become a dominant configuration for high‐performance PSCs, owing to breakthrough in self‐assembling monolayer (SAM)‐like HTL [14], which mitigates the instability issue of Spiro‐OMeTAD used as the HTL of conventional n‐i‐p PSCs [15]. Until now, few publications have focused on p‐i‐n C‐PSCs due to the energy‐level mismatch between electron transport layers (ETLs) and carbon electrode and mechanical damage to a thin ETL by the carbon paste processing [2]. Despite the challenge, there have been several attempts to adopt carbon electrode in p‐i‐n PSCs. Babu et al. inserted a 5 nm‐thick evaporated Cr layer as a buffer layer in p‐i‐n C‐PSCs, reaching PCE of up to 14.5% [8]. Lukas et al. used the following configuration: [6, 6]‐phenyl‐C_61_‐butyric acid methyl ester (PCBM)/SnO_2_/PEDOT:PSS (PH1000)/carbon to tune the interfacial energetics to achieve 16.1% [16]. A hierarchical energy‐level‐aligned interface of C_60_ and atomic‐layer‐deposited (ALD)‐SnO*_x_* bilayer was used to achieve 21.1% [17]. Príncipe et al. adapted carbon paper to achieve 15.6% [18], although an Au:MoO_3_ thin layer was required to improve the energy alignments.

In contrast to a simple modification of p‐i‐n C‐PSCs by inserting a thin layer beneath the carbon electrode, in the n‐i‐p C‐PSCs a number of HTM additives in carbon paste have been explored to tune its work function, including Mn_3_O_4_ [19], NiO [20], and WO_3_ [21]. Nonetheless, analogous additive engineering of carbon paste for p‐i‐n C‐PSCs has been limited. Therefore, electron transport materials (ETMs) such as fullerene derivatives [22], organic materials [23, 24], ZnO [25], SnO_2_ [26], TiO_2_ [27], Zn_2_SnO_4_ [28], and WO_3_ [29] are considered as the additives for carbon paste of p‐i‐n C‐PSCs. However, many of them are sensitive to process environment, high‐cost, and/or require solution process and high‐temperature sintering. Cubic silicon carbide (3C‐SiC) has been proposed in simulation studies as an ETM for PSCs due to its excellent energy‐level matching of conduction band minimum (CBM, −3.8 eV) with that of lead halide perovskites [30, 31, 32, 33, 34, 35, 36]. Although 3C‐SiC is highly affordable and used in many industries [37], it is chemically inert and not easy to integrate into PSCs using conventional wet‐chemistry techniques. To overcome inertness, 3C‐SiC was functionalized with sodium dodecylsulfate (SDS) [38], cetyltrimethylammonium bromide (CTAB) [39], and 3‐aminopropyltriethoxysilane (APTES) [40]. The triethoxysilane head of APTES can form strong Si‐O‐Si bonding to SiC‐OH [40], and the amino terminal can act as a universal linker. APTES forms a dense, ordered, covalently bound SAM on both Si‐ and C‐sites of SiC. Fu et al. found that APTES‐modified SiC was oil‐compatible and could improve the insulating oil performance [41]. Guevara‐Lora et al. reported that APTES modification significantly enhanced the protein binding to the SiC nanopowder [42]. APTES was also used for surface passivation of SiO_2_ gate dielectric for perovskite thin‐film field‐effect transistors [43]. Wang et al. used APTES for improving the affinity of perovskite thin films with SiO_2_ substrate [44]. Moreover, 3‐aminopropyltrimethoxysilane (APTMS), which has three methoxy groups instead of three ethoxy groups as in APTES, was used as a passivator for perovskite layer in PSCs [45].

Here, we introduce APTES‐functionalized 3C‐SiC nanoparticles as a novel, low‐cost, multifunctional additive for home‐made carbon paste for p‐i‐n C‐PSCs. The shallower CBM of SiC than the Fermi level of carbon electrode could create a Schottky‐like contact, which prevents electrons from being trapped in SiC nanoparticles in carbon paste [46, 47]. Most importantly, we found that APTES functionalization simultaneously (i) improves the rheology of the carbon paste for void‐free film formation and (ii) enables intimate contact with the underlying C_60_ ETL. With this simple additive, we achieve a champion PCE of 18.43% (stabilized 18.2%)—among the highest reported for p‐i‐n C‐PSCs—accompanied by small hysteresis and excellent ambient stability.

## 2 Results and Discussion

### 2.1 Effect of APTES on SiC powder

**FIGURE 1:**
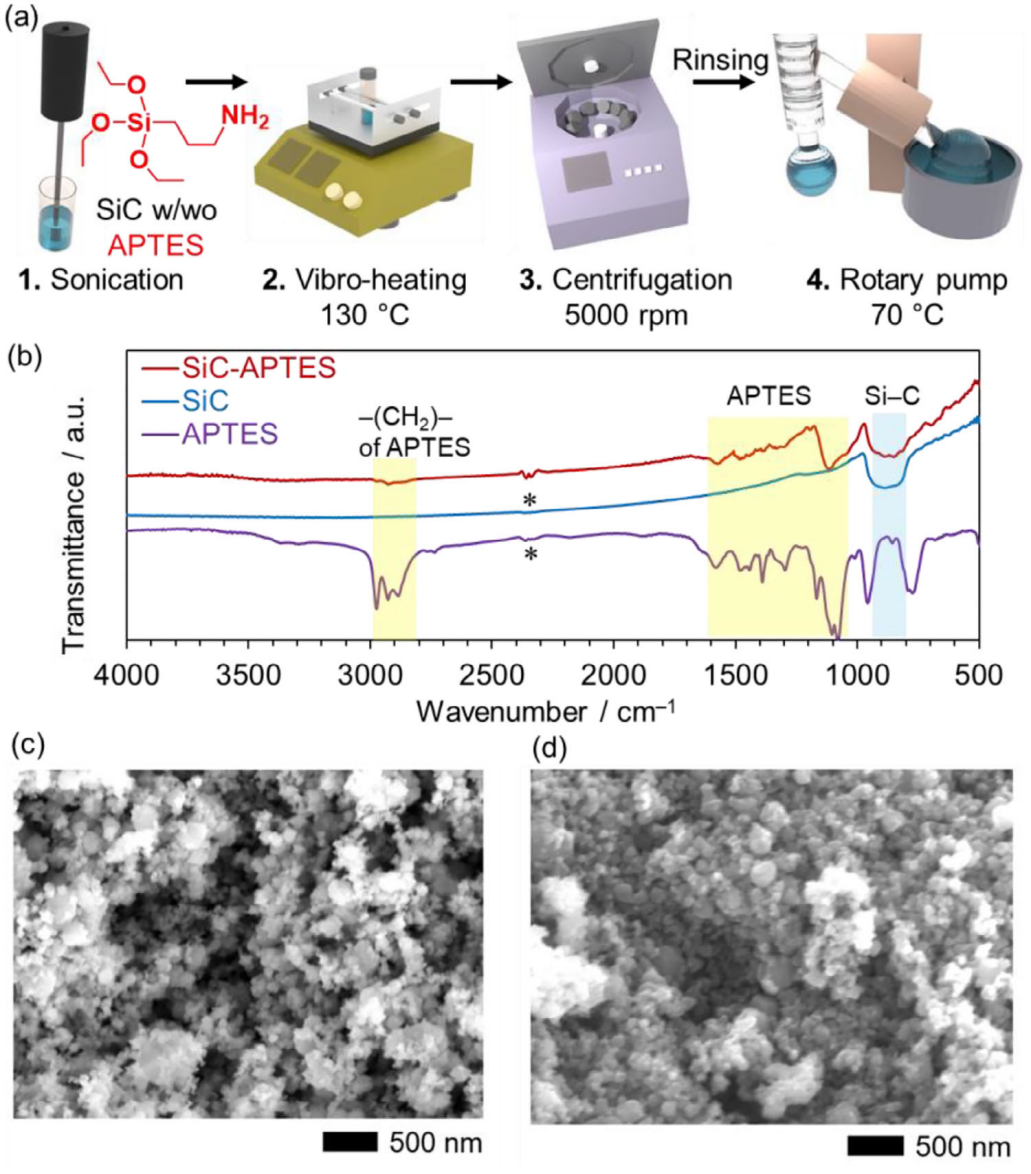
(a) Diagram illustrating modification of SiC powder (step 1 to step 4). The chemical structure of APTES is drawn. (b) FTIR spectra of SiC, bare APTES, and SiC‐APTES. The yellow and blue regions are attributed to APTES and SiC, respectively. The asterisk is an instrumental peak. SEM images of (c) SiC powder and (d) SiC‐APTES powder.

The modification of 3C‐SiC with APTES involves a two‐cycle process, where a schematic of one cycle is shown in Figure 1a. The first cycle is to hydroxylate the surface of SiC, which starts with alkaline activation of SiC nanoparticles in KOH aqueous solution. After ultrasonic dispersion (step 1), the suspension was subjected to vibro‐heating for 90 min using a home‐made system shown in Figure S1 (step 2). This vibro‐heating system provides simultaneous mechanical agitation and heat treatment, offering superior hydroxyl‐group generation compared to conventional UV‐ozone treatment as seen from the comparison of the Fourier transform infrared (FTIR) spectra (Figure S2). The enhanced OH density arises from more efficient removal of the native oxide layer and subsequent nucleophilic attack by OH^−^ on Si─C bonds, generating abundant silanol (Si‐OH) sites. Following this reaction, powder was centrifuged and rinsed with deionized water twice to eliminate residual KOH (step 3). Then the powder was dispersed in isopropylalcohol (IPA), and the solvent was removed using a rotary evaporator (step 4). The recovered SiC‐OH powder exhibited a pronounced broad absorption band centered at ∼3400 cm^−1^ in the FTIR spectrum (Figure S2), assigned to O─H stretching vibrations of surface hydroxyl groups [42]. This intense band confirms successful surface hydroxylation, which is a prerequisite for subsequent silanization.

The second cycle to modify SiC‐OH with APTES was done similarly to the process described above. The hydroxylated SiC‐OH was redispersed by sonication in 1‐butanol, followed by sequential addition of anhydrous *N*,*N*‐dimethylformamide (DMF) and APTES (step 1). Anhydrous DMF serves multiple roles: (i) improving the solubility of APTES, (ii) acting as a water scavenger, and (iii) facilitating uniform mixing in the low‐water environment. The mixture was then placed on the vibro‐heating reactor for the reaction between the surface Si‐OH and APTES, forming covalent Si‐O‐Si linkages (step 2). The unreacted byproducts were removed by centrifugation and washing with isopropanol (step 3). Then the solvent was removed, yielding the final SiC‐APTES powder (step 4).

FTIR analysis of SiC‐APTES (Figure 1b) revealed new characteristic bands attributable to the APTES layer. The band at 800–950 cm^−1^ is the stretching vibration of Si─C bond [41]. For the modified SiC‐APTES, the infrared absorption peaks at 2800–3000 cm^−1^ are symmetric stretching vibration peaks of methylene (─CH_2_─) [41, 42]. In addition, the correspondence of the characteristic peaks in the fingerprint region at 1000–1600 cm^−1^ in both bare APTES and SiC‐APTES spectra confirm the successful incorporation of APTES onto the SiC surface. The x‐ray diffraction (XRD) pattern of SiC‐APTES powder shows no additional peaks or shift in position confirming that APTES only modified the surface of SiC particles (Figure S3). However, the SiC powder after modification becomes visibly darker (Figure S4), probably because the organosilane monolayer changes the refractive index at the surface. These spectral features suggest successful covalent attachment of APTES. Photoelectron yield spectroscopy (PYS) measurements show similar valence band maximum (VBM) at around −7.1 eV regardless of APTES functionalization (Figure S5). Scanning electron microscopy (SEM) analysis (Figure 1c,d) revealed a moderate reduction in average diameter from ∼144 ± 5 nm (pristine SiC) to ∼121 ± 5 nm after complete functionalization (digitized SEM images are in Figure S6). This decrease can be attributed to ultrasonication (step 1 in Figure 1a), which efficiently disrupts agglomerates in as‐received samples.

### 2.2 Effect of SiC‐APTES on Carbon Paste

**FIGURE 2:**
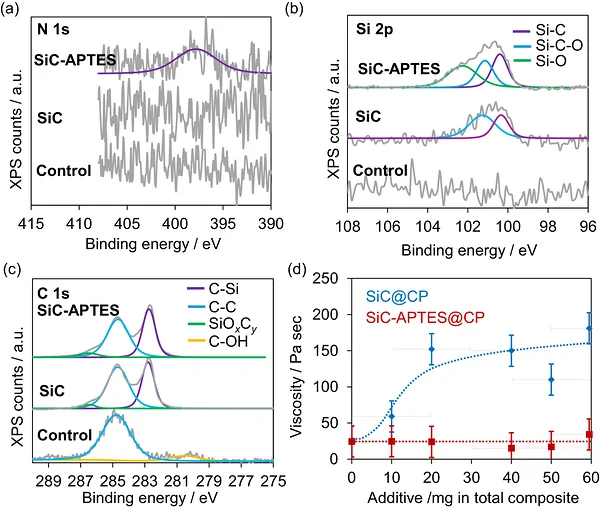
XPS: (a) N 1s, (b) Si 2p, and (c) C 1s of carbon electrode (control) with SiC or SiC‐APTES (30 mg in total 373 mg carbon composite). (d) Viscosity of the carbon paste with/without SiC or SiC‐APTES additives. The error bars are based on the standard deviation of the control (0 mg) samples. The dotted lines are eye‐guides.

Carbon paste was prepared as described in Experimental. In short, SiC or SiC‐APTES powders were added to the carbon paste during the ball milling process. Figure 2a shows high‐ resolution x‐ray photoelectron spectroscopy (XPS) N 1s spectra of control carbon paste deposited on fluorine‐doped tin oxide (FTO) with SiC or SiC‐APTES additive. The presence of the N 1s peak after functionalization is an indicator of successful attachment of APTES to SiC [40, 48, 49].

Both samples with addition of SiC or SiC‐APTES contain Si─C and Si─C─O groups on the XPS Si 2p scan (Figure 2b), while these are absent for the control carbon paste sample. As for the C scan (Figure 2c), we identify additional peaks in SiC‐containing samples as C─Si bonds and SiO*_x_* C*_y_*, while the control sample includes a weak C─OH peak (in accordance with O 1s scan). Detailed XPS analysis of these and O 1s scan (Figure S7) with Supporting Note S1 is provided in Supporting Information.

Visually, the paste becomes very viscous after adding SiC powder, but not SiC‐APTES. It is confirmed by viscosity measurements shown in Figure 2d (the original time‐dependent viscosity curves and data are shown in Figure S8a and Table S1, respectively). The viscosity of the control carbon paste (24 Pa·s) is an order of magnitude lower than ones reported in the literature at the same shear rate [19]. This may be related to the addition of 0.3 mL of IPA after grinding as mentioned in the experimental section. The addition of pristine SiC nanoparticles to the carbon paste leads to a dramatic increase in viscosity. As drawn by the blue line in Figure 2d, the viscosity reaches over 150 Pa·s at loadings above 20 mg per total 373 mg carbon composite (5.4 wt%, see Experimental). This sharp rise is attributed to chemically inert surface of pristine SiC, which causes extensive nanoparticle agglomeration. These large agglomerates interlock with the carbon matrix, forming a jammed, highly concentrated particulate network that resists shear, thereby producing a steep increase in viscosity. In striking contrast, APTES‐functionalized SiC (SiC‐APTES) maintains the paste viscosity below 30 Pa·s across the entire loading range studied, yielding a smooth, easily spreadable formulation even at the highest additions (60 mg, 16 wt%). This results from surface‐bound amine/silane groups of APTES, which increase affinity with the carbon paste components and introduce steric repulsion between particles. Unlike pristine SiC, SiC‐APTES resists aggregation so that the paste remains flowable. Surface functionalization of SiC with APTES transforms an otherwise problematic high‐viscosity chemically inert filler into an effective multifunctional additive for carbon electrodes in p‐i‐n C‐PSCs.

### 2.3 Effect of SiC and SiC‐APTES Additives to Carbon Paste on PSCs

**FIGURE 3:**
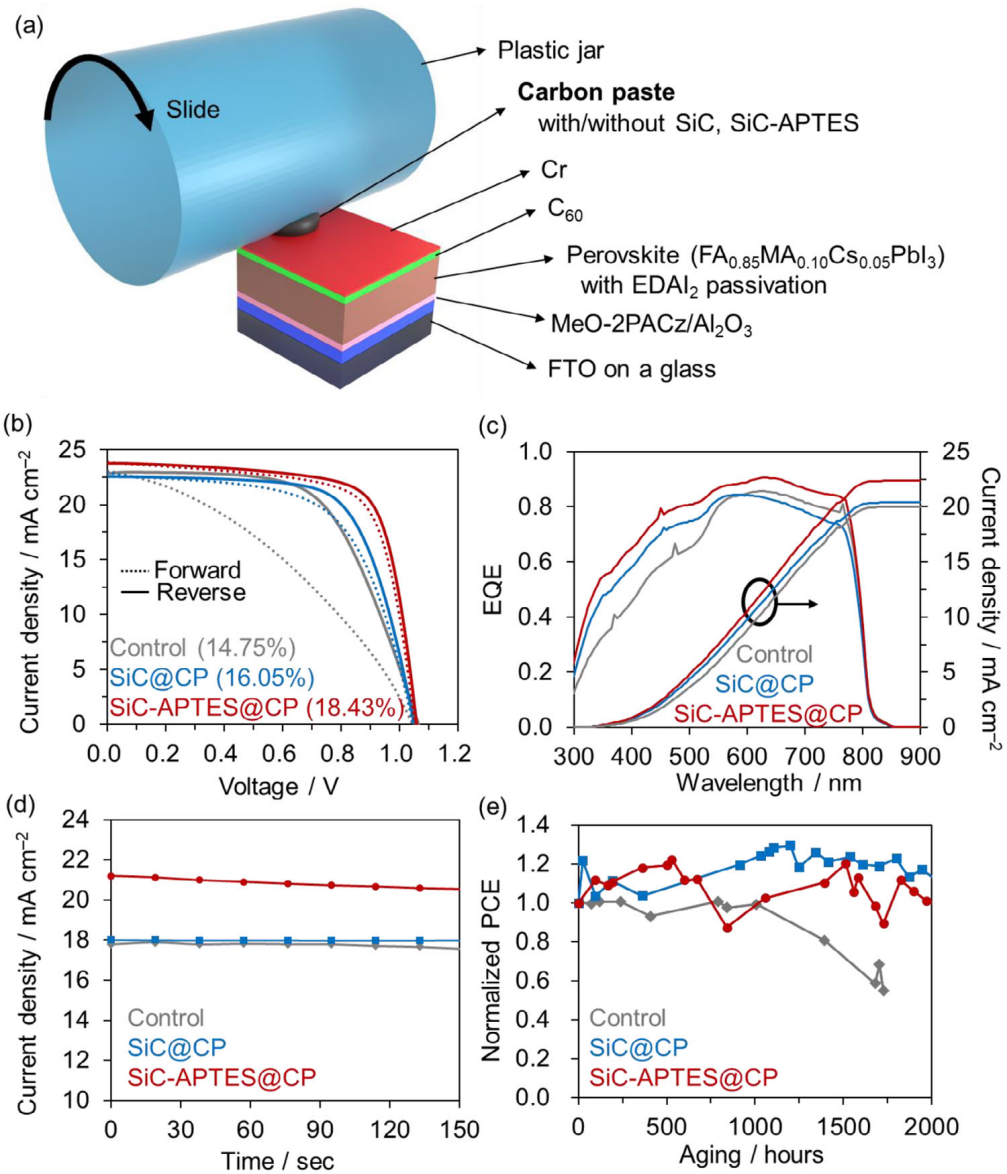
(a) Schematic of the device structure of p‐i‐n C‐PSC. (b) *J–V* curves of the best devices prepared without (control) and with addition of SiC (30 mg in total 373 mg carbon composite) or SiC‐APTES (10 mg). (c) EQE spectra and integrated short‐circuit current density. (d) Steady‐state photocurrent measurements (measured at 0.61 V for control PSCs, and 0.73 V and 0.86 V for SiC and SiC‐APTES carbon pastes PSCs, respectively). (e) Stability tests of C‐PSCs stored at 20°C–25°C and 40%–60% RH.

The impact of pristine SiC and APTES‐functionalized SiC additives on the photovoltaic performance of inverted (p‐i‐n) C‐PSCs was systematically investigated. As shown in Figure 3a, the device architecture was FTO/MeO‐2PACz/Al_2_O_3_/FA_0.85_MA_0.1_Cs_0.05_PbI_3_/C_60_/Cr/carbon with/without SiC or SiC‐APTES (FA: formamidinium, MA: methylammonium). The carbon electrode was deposited by sliding a plastic jar on the device upon a tape mask (see Experimental). As shown in Figure 3b, the control device (pure carbon paste: CP) delivered a champion PCE of 14.75% with open‐circuit voltage (*V*_OC_) = 1.059 V, short‐circuit current density (*J*_SC_) = 21.52 mA cm^−2^, and fill factor (FF) = 64.6% for the reverse scan (statistics are listed in Table 1). Incorporating unmodified SiC (SiC@CP at the optimal amount, vide infra) slightly improved *J*_SC_ and FF, yielding a best PCE of 16.05% (consistent with the poor paste rheology and resulting in inhomogeneous carbon films). Even though SiC‐added carbon paste is more viscous, it still delivers a noticeable PCE gain. This proves that SiC itself is intrinsically beneficial, probably due to high affinity of SiC to the C_60_ ETL and electron transfer from SiC to carbon [46]. In stark contrast, the SiC‐APTES (SiC‐APTES@CP at the optimal amount, vide infra) device exhibited a champion PCE of 18.43%, with the significantly improved *J*_SC_ = 23.78 mA cm^−2^ and FF = 73.2%. Hysteresis was eliminated with SiC‐APTES (hysteresis index: HI = 0.04) versus 0.36 for control, signifying balanced electron/hole transport and suppressed ion migration/defect‐mediated trapping at the interface. The most pronounced improvements are observed in FF and in both series (*R*_s_) and shunt resistance (*R*_sh_), which directly govern FF in PSCs. Specifically, *R*_s_ decreased from 9.86 Ω·cm^2^ (control) to 3.16 Ω·cm^2^ (SiC‐APTES), indicating reduced interfacial resistance and improved charge transport. Meanwhile, *R*_sh_ increased from 338 to 694 Ω·cm^2^, suggesting suppressed leakage pathways. The simultaneous decrease in *R*_s_ and increase in *R*_sh_ confirm improved diode quality and interfacial contact in the SiC‐APTES‐modified devices. Dark *J*–*V* curves (Figure S8b) provide additional insight into recombination suppression. SiC‐APTES device has the lowest dark current across the entire reverse bias range (*V* < 0), nearly 1 order lower than control device at −1 V. This indicates effective suppression of shunt pathways and recombination, consistent with the increased *R*_sh_ observed in the SiC‐APTES device. Electrochemical impedance spectroscopy (EIS) measurements also support the largest recombination resistance for the SiC‐APTES device (Figure S9). Interestingly, Mott‐Schottky analyses demonstrate one‐order smaller carrier trap density in SiC‐APTES device (6.28 × 10^15^ cm^−3^) than those in the control and SiC‐based devices (4.26–4.34 × 10^16^ cm^−3^) (Figure S10).

**TABLE 1:** Summary of C‐PSC (FTO/MeO‐2PACz/Al_2_O_3_/FA_0.85_MA_0.1_Cs_0.05_PbI_3_/C_60_/Cr/Carbon) performance.^a^

Carbon paste	PCE [%]	*J*_SC_ [mA cm^−2^]	*V*_OC_ [V]	FF	*R*_s_ [Ω cm^2^]	*R*_sh_ [Ω cm^2^]	HI^b^
Control	14.75 (12.49 ± 0.98)	21.52 (19.94 ± 0.92)	1.059 (1.059 ± 0.031)	0.646 (0.593 ± 0.032)	9.86 (8.39 ± 1.92)	338 (232 ± 86)	0.36 (0.40 ± 0.12)
SiC	16.05 (14.25 ± 0.80)	22.55 (21.82 ± 0.90)	1.050 (1.053 ± 0.009)	0.677 (0.621 ± 0.027)	6.27 (8.10 ± 1.61)	906 (436 ± 215)	0.09 (0.22 ± 0.12)
SiC‐APTES	18.43 (17.09 ± 0.91)	23.78 (23.67 ± 0.69)	1.058 (1.050 ± 0.016)	0.732 (0.682 ± 0.079)	3.16 (4.94 ± 1.45)	694 (487 ± 151)	0.04 (0.09 ± 0.08)

^a^ Under pseudo sunlight (100 mW cm^−2^). The number of devices is more than 8 for each. The values are the maximum PCE and their device parameters in the reverse scan. The values in the parentheses are the average and standard deviation. The optimal amounts of SiC and SiC‐APTES are 30 and 10 mg per total 373 mg of the carbon paste composite, respectively (corresponding to 8.0 and 2.7 wt%, respectively).

^b^ Hysteresis index = (reverse PCE – forward PCE)(reverse PCE)^−1^.

External quantum efficiency (EQE) spectra (Figure 3c) exhibit improved charge collection regardless of the wavelength. The integrated *J*_SC_ values from the EQE spectra are 19.99 mA cm^−2^ for control, 20.39 mA cm^−2^ for SiC, and 22.35 mA cm^−2^ for SiC‐APTES PSCs, in agreement with the performance statistics (Table 1). Steady‐state photocurrent under continuous 1‐sun illumination for 150 s (Figure 3d) revealed stable output for all devices. Unencapsulated stability tests under 40%–60% RH (Figure 3e) showed the PCE of the control device drops to 54% after 1700 h, while SiC and SiC‐APTES devices retain >90% PCE after this period of time (Tables S2–S4). This enhanced stability is consistent with previous reports on carbon‐electrode‐based perovskite devices, where interfacial engineering suppresses ion migration and improves operational durability [50, 51].

The observed fluctuations in the stability traces are primarily attributed to storage and measurements performed under ambient laboratory conditions with varying humidity and temperature (20°–25°C, 40%–60% RH). Such conditions can induce transient physicochemical changes in perovskite films, including ion redistribution, partial PbI_2_‐related passivation at interfaces and grain boundaries, and gradual evaporation or redistribution of residual solvents, resulting in non‐monotonic variations in charge transport and device performance over time [52]. In addition, fluctuations arise from measurement variability due to the large carbon electrode area (Figure S11). The smaller measurement mask cannot be precisely repositioned on the same spot each time, leading to inconsistent effective active areas.

**FIGURE 4:**
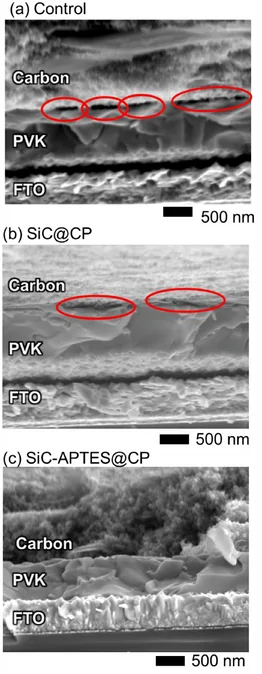
Cross‐sectional SEM images of PSCs made with carbon paste: (a) Control, (b) with SiC, and (c) with SiC‐APTES. PVK represents perovskite. The red circles indicate the observed voids at the interface.

To further understand the difference in the performance, cross‐sectional SEM images of PSCs were taken (Figure 4). The control carbon layer (Figure 4a) exhibits large voids and poor adhesion to the underlying layers (circled in red). Upon adding SiC to the paste, the amount of voids decreases (Figure 4b) due to affinity of the carbon paste to C_60_, consistent with the decreased *R*_s_ compared to the control devices. Similar improvements in interfacial contact and void suppression in carbon‐based p‐i‐n architectures have been widely reported for nanoparticle‐modified carbon electrodes [53, 54, 55]. Well‐dispersed SiC‐APTES‐containing paste (Figure 4c) shows the superior film‐forming ability and intimate interfacial contact enabled by the low‐viscosity and high affinity to the underlying layers. The macroscopic cross‐sectional images are shown in Figure S12. The thickness (∼12 µm) and surface morphology of carbon electrode are unchanged by the additives, but the cross‐sectional structure of the carbon layer is improved by adding SiC or SiC‐APTES. In particular, the SiC‐APTES‐containing carbon films look denser, smoother, and more uniform than the control.

**FIGURE 5:**
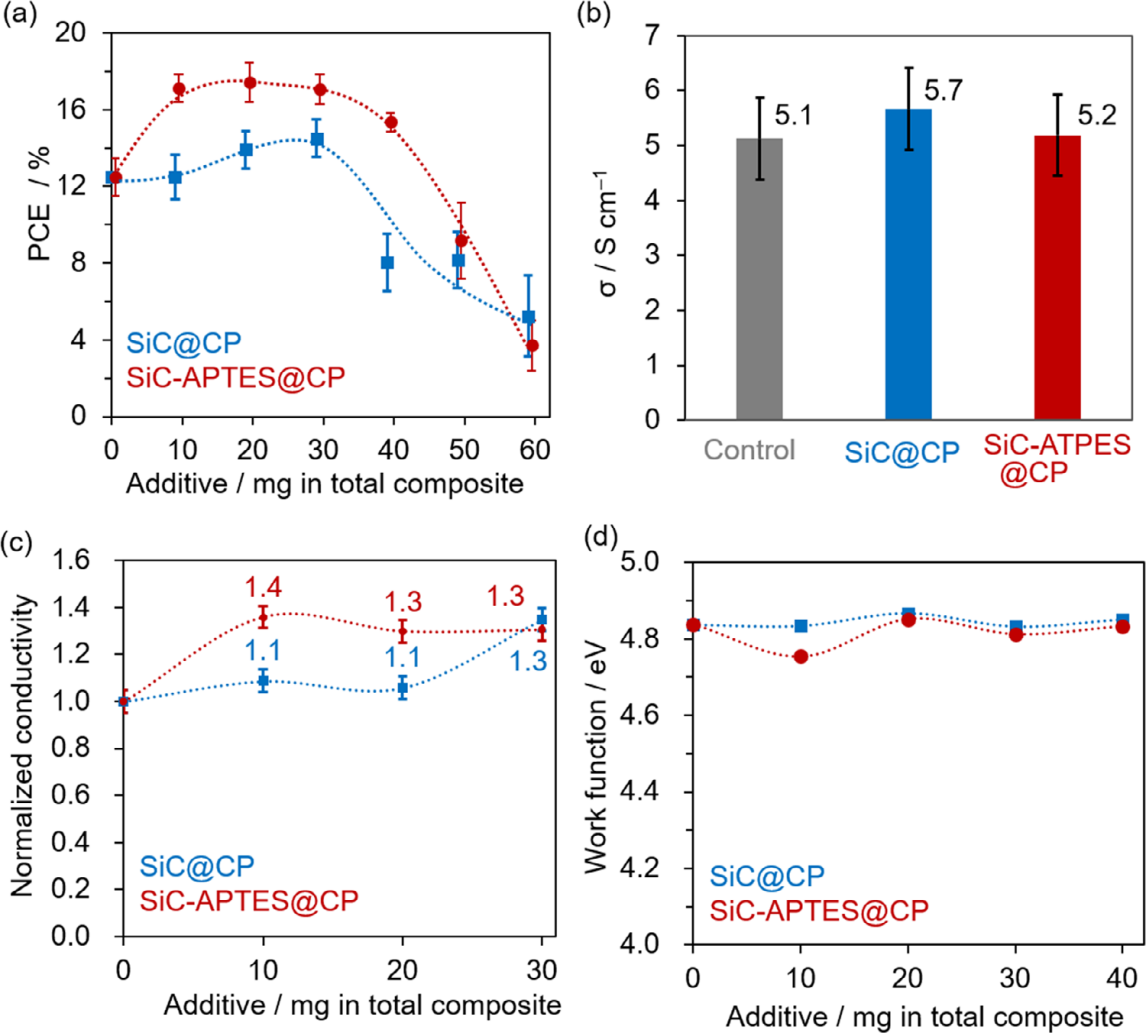
(a) Average PCE of p‐i‐n C‐PSCs vs. SiC or SiC‐APTES content (mg) in carbon paste (total 373 mg). Error bars: standard deviation. (b) Bulk conductivity of carbon electrode measured by a four‐probe method. The error bar is based on the experimental deviation of control samples. (c) Normalized conductivities extracted from *J–V* curves of the samples with structure: ITO/C_60_ (20 nm)/Cr (5 nm)/Carbon w/wo additives. The error bar is based on the experimental deviation of control samples. The dotted lines are eye‐guides. (d) KP surface potential of carbon paste w/wo additives.

The simultaneous improvements in *J*_SC_, FF, and hysteresis can be explained by beneficial roles of SiC‐APTES due to enhanced interfacial adhesion to C_60_ (the alkyl chains of APTES dramatically lower the surface energy mismatch between hydrophilic SiC and hydrophobic C_60_) and rheological optimization enabling high‐quality, void‐free carbon electrodes. To determine the optimal concentration of the functionalized additive, carbon pastes were prepared with different loading of SiC or SiC‐APTES ranging from 0 mg (pure carbon control) to 60 mg per total 373 mg of the carbon paste composite (16 wt%). The average PCEs of the resulting p‐i‐n devices are summarized in Figure 5a (the values are listed in Tables S5 and S6). For unmodified SiC, the average PCE gradually increases from 12.5% (0 mg) to a modest maximum of 13.3%–14.5% at 20–30 mg loading, then slowly declines at higher concentrations. This steady upward trend unambiguously confirms that 3C‐SiC nanoparticles are beneficial even without surface functionalization. For SiC‐APTES loading, the average PCEs exhibit a clear volcano‐type dependence, where they rise steeply with initial addition of SiC‐APTES, reaching plateau values of 17.1%–17.4% between 10 and 30 mg. The champion device at 10 mg loading (2.7 wt%) delivered the maximum PCE = 18.43%. Beyond 30 mg, the PCE gradually declines, dropping sharply above 40 mg, and devices become highly shunted and barely functional at 60 mg because an excessive amount of APTES insulator may lead to an increase in the series resistance. Although SiC‐APTES exhibits markedly better rheology and dispersion than pristine SiC, both additives display a similar optimal loading range between 10 and 30 mg. This indicates that the primary performance enhancement arises from improved interfacial contact with the C_60_ layer due to the high intrinsic affinity of SiC nanoparticles, rather than from bulk rheological effects alone.

To elucidate the influence of the additives on the electric properties of carbon electrodes, we fabricated two types of test structures: 1) carbon pastes on glass to measure bulk conductivity by four probe measurements and 2) indium tin oxide (ITO)/C_60_ (20 nm)/Cr (5 nm)/carbon to extract interfacial charge‐transport resistance. As shown in Figure 5b, the bulk conductivities of carbon paste are not altered much after addition of SiC or SiC‐APTES and stay at the level of ∼5 S cm^−1^ (in accordance with the literature [56]). In contrast, the interfacial conductivity (or resistance mainly at the C_60_/Cr/Carbon interface) (Figure 5c, Supporting Note S2) shows a good correlation with the PCE trend observed in the concentration dependence of SiC and SiC‐APTES (the current‐voltage curves and device pictures are provided in Figure S11). While the normalized conductivity does not change for SiC additive at 10–20 mg loading, it increases at the loading of 30 mg and corresponds with the maximum PCE achieved. Similarly, SiC‐APTES addition leads to an increase in interfacial conductivity after just adding 10 mg to the paste, which again correlates well with PCE. This measurement also confirms that the optimal loading window is remarkably wide with SiC‐APTES (10–30 mg, 2.7–8.0 wt%). This substantial reduction in contact resistance is the primary origin of the observed PCE boost that is in accordance with the intimate contact of ETL and carbon electrode observed in SEM.

Another possible reason is the change in work function of SiC and SiC‐APTES‐loaded carbon electrodes. Contrary to the SCAPS‐1D simulations [30, 31, 32, 33, 34], our Kelvin probe (KP) measurements show no detectable change in work function between 4.75 and 4.87 eV compared to the pristine carbon (4.84 eV) relative to the gold reference (assumed to be 4.80 eV, Figure S13). The work functions measured by PYS were also similar within the experimental deviations (5.43 to 5.60 eV, Figure S14). This is because the carbon electrode is highly conductive, while SiC is highly resistive. In addition, the volume fraction of SiC and SiC‐APTES (16 wt% at the maximum) is still too low to dominate the surface electronic structure.

Although the anticipated work function tuning of the carbon electrode was not observed, the passivation effect needs to be examined, as analogous APTMS has been used as a passivator of PSCs [45]. We added APTES directly to the paste at 0.7 and 3 wt%, but the device performance decreased to 13.4% and 1.5%, respectively (Figure S15). This severe degradation can be attributed to the high reactivity of neat APTES, which can chemically damage the underlying layers during deposition and annealing. In contrast, anchoring APTES onto SiC nanoparticles fully suppresses these detrimental side reactions, making SiC‐APTES the preferred and safe additive for carbon‐electrode p‐i‐n PSCs.

## 3 Conclusions

We demonstrate that APTES‐functionalized cubic SiC nanoparticles serve as an effective, low‐cost multifunctional additive for carbon electrodes in inverted (p‐i‐n) PSCs. APTES modification converts inert, highly agglomerating SiC into a well‐dispersed filler that dramatically improves carbon paste rheology, promotes void‐free contact, and reduces interfacial resistance. As a result, the SiC‐APTES carbon electrode improved device performance including reduced series resistance, suppressed leakage current, nearly hysteresis‐free operation, and enhanced charge collection, although the work functions and bulk conductivities of carbon pastes were mostly unchanged. We demonstrate that the p‐i‐n C‐PSCs reach the maximum PCE of 18.43% (compared to 14.75% for the control device), among the highest reported for this architecture, with excellent operational stability.

## 4 Experimental Section

### 4.1 Modification of SiC with APTES

SiC (50 nm, β form, Wako–Fujifilm) nanoparticles (200 mg) were activated in a 0.5 M KOH aqueous solution. Then, after ultrasonic dispersion for 30 min (step 1 in Figure 1a), the suspension was transferred to a home‐built vibro‐heating reactor (Figure S1) and treated at 130°C for 90 min (step 2 in Figure 1a). Following reaction, to eliminate the residual KOH, the powder was centrifuged and rinsed with deionized water twice (step 3 in Figure 1a). The powder was redispersed in IPA and the solvent was removed using a rotary evaporator (70°C, 200 kPa for 10 min, then 50 kPa for an additional 10 min) (step 4 in Figure 1a). The recovered SiC‐OH powder (typical yield ≈ 180 mg, 90%) was modified with APTES similarly to the described above process. Firstly, the hydroxylated SiC‐OH was redispersed by sonication in 3 mL of 1‐butanol, followed by sequential addition of 1 mL anhydrous DMF and 1 mL APTES. The mixture was again processed in the vibro‐heating reactor for 90 min to promote hydrolysis of ethoxy groups and subsequent condensation with surface Si‐OH, forming covalent Si‐O‐Si linkages. After reaction, excess silane and byproducts were removed by repeated centrifugation and washing with IPA. Drying under identical rotary‐evaporation conditions yielded the final SiC‐APTES powder. These nanoparticles were added to the carbon paste.

### 4.2 Preparation of Carbon Electrode

To prepare carbon paste for the electrode deposition, 116 mg of ethylcellulose (80–120 cps, Nacalai Tesque), 186 mg of graphite (>98%, Wako–Fujifilm), 71 mg of carbon black (ECP600JD, Lion Specialty Chemicals), and 1.6 mL of butanol (anhydrous, Wako–Fujifilm) [16] were put into a grinding stainless vessel with volume of 5 mL and two stainless steel balls with *φ*  = 7 mm. The ball milling process was carried out using a Mixer Mill MM400 (Retsch) at 30 Hz for 8 h followed by 1 more hour after adding 0.3 mL IPA. For the carbon pastes with additives, different amounts of SiC or SiC‐APTES were added to the vessel and grinded together with other components.

### 4.3 Preparation of Perovskite Electrode

A 1.5 M perovskite solution with the formula FA_0.85_MA_0.10_Cs_0.05_PbI_3_ was prepared by dissolving 12.66 mg MACl (>99.99%, Greatcell Solar Materials: GSM), 19.49 mg CsI (>99.99%, Sigma‐Aldrich), 219.26 mg FAI (>99.99%, GSM), 23.85 mg MAI (>99.99%, GSM), 20.86 mg PbCl_2_ (>99.5%, Tokyo Chemical Inc.: TCI), and 760.67 mg PbI_2_ (>99.99%, TCI) in 1 mL of DMF: dimethylsulfoxide (DMSO) (4:1 v/v) solvents (super dehydrated, Wako–Fujifilm) [57] and stirred at 50°C for more than 2 h in a N_2_‐filled glovebox (H_2_O<0.1 ppm, O_2_<0.1 ppm).

### 4.4 Device Fabrication

FTO on glass substrates (8 Ω/sq, Sigma‐Aldrich) were patterned by etching with HCl and Zn to prevent shunting. The substrates were then sonicated for 15 min in DI water with 10% cleaning liquid (USC‐702(SND)), rinsed with DI water, acetone, and sonicated again in IPA for 15 min. All operations below were done in a N_2_‐filled glovebox. To prepare an HTL, a 1.5 mM MeO‐2PACz (>98%, TCI) solution in ethanol (super dehydrated, Wako–Fujifilm) was spin‐coated on the substrates and annealed at 100°C for 15 min. MeO‐2PACz layers were passivated with Al_2_O_3_ by spin‐coating the 20 wt% nanoparticle dispersion (Sigma‐Aldrich)(diluted 40 times with IPA) at 3000 rpm for 30 s, followed by annealing for 10 min at 100°C. Then, the perovskite solution (100 µL) was spin‐coated onto HTL at 1000 rpm for 10 s, then at 5000 rpm for 40 s. After 51 s of the spin‐coating, 200 µL of chlorobenzene (Sigma‐Aldrich, 0.005% water) dripped onto the center of the substrate. The films were annealed at 100°C for 15 min. Next, the perovskite layers were passivated with 1.6 mM solution of ethylenediamine diiodide (EDAI_2_) (>98%, Sigma‐Aldrich) in a 1:1 volume mixture of anhydrous IPA (>99.5%, Wako‐Fujifilm) and super anhydrous toluene (Wako‐Fujifilm). It was spin‐coated onto the perovskite film, followed by annealing at 100°C for 10 min [58]. For the ETL, 20 nm of C_60_ (>99.5%, TCI) and 5 nm of Cr (>99.99%, Nilaco) were thermally evaporated onto the perovskite films at a rate of 0.1 Å s^−1^ under a vacuum of ∼10^−5^ Pa. The electrode pattern was achieved by manual masking with a scotch tape. The carbon electrode was manually bar‐coated using a plastic jar (diameter = 55 mm, height = 110 mm) four times and then the device was annealed at 80°C for 10 min.

### 4.5 Characterization

The light mask size was 0.08 cm^2^. The scan was conducted from 0 to 1.2 V (forward) and then reverse, with a step size of 0.02 V, a hold time of 150 ms, an integration time of 150 ms, and a measurement delay of 100 ms. All the measurements were performed in a N_2_‐filled glovebox. Current density‐voltage curves were measured using a source meter unit (SMU) (Keysight B2910BL) under AM1.5G pseudo‐solar illumination at 100 mW cm^−2^ using an LED solar simulator (LumiSun‐50, Innovation in Optics Inc. USA, IEC class A+A+A). The output powers of the solar simulators were monitored by a calibrated silicon standard cell of Bunko Keiki BS‐501BK with a Sigma‐Koki Inc. HAF‐50H heat absorbing short‐pass filter. The output of the standard cell with the filter was calibrated by EQE measurements. The conductivity measurements were performed using the same instruments with those of solar cell characterization in dark. The four probe measurements were conducted using a Mitsubishi Chemical Loresta PSP prober. EIS measurements were performed on a HIOKI IM3570. SEM was performed on a field emission SEM JEOL JSM‐IT700HR at 5 keV. The grain size of perovskite was evaluated using MorphoLibJ library in ImageJ. PYS experiments were carried out in a vacuum (<10^−2^ Pa) using a Bunko Keiki BIP‐KV201 photoemission spectrometer (extraction voltage 10 V). Viscosity measurements were performed using a parallel‐plate geometry on an Anton Paar MCR302 rheometer at a shear rate of 0.1 s^−1^ and a temperature of 25°C (gap: 0.5 mm). Work functions of the carbon electrodes were measured in a nitrogen‐filled glovebox in the dark using a Kelvin probe analyzer (KP technology) equipped with a 2 mm gold tip. The work function was referenced to that of the gold substrate (4.8 eV) before each measurement. XPS analyses were conducted using a KRATOS ULTRA2 photoelectron analyzer under a base pressure of approximately 10^−7^ Pa using monochromatized Al‐Kα radiation (*hν* = 1486.6 eV) at an operational power of 300 W (15 kV, 20 mA). EQE spectra were recorded using a Bunko Keiki model SM‐250KD. FTIR spectra were recorded using a Jasco FT/IR‐4700AC spectrometer. Powder XRD measurements were conducted using a Rigaku MiniFlex‐600 instrument using Cu‐Kα radiation (*λ* = 1.54 Å).

## Conflicts of Interest

The authors declare no conflicts of interest.

## Supporting information




**Supporting File**: smll74393‐sup‐0001‐SuppMat.pdf.

## Data Availability

The data that support the findings of this study are available from the corresponding author upon reasonable request.
