# Supplementary material for: Improved Charge Transport and Device Performance in p‐i‐n Carbon Electrode Perovskite Solar Cells Using SiC‐APTES Additive
Source: Small. 2026 Jul 6;22(48):e74393. doi: 10.1002/smll.74393 (PMC13509056; doi:10.1002/smll.74393)
Supplement: Supplementary file 1 [file SMLL-22-e74393-s001.pdf]

Supporting Information

Improved Charge Transport and Device Performance in p-i-n Carbon Electrode Perovskite Solar Cells Using SiC-APTES Additive

*Mikhail Pylnev[1,2], Ryosuke Nishikubo[1,3], Tomoya Nakamura[4], Ryoji Shimomura,[1] Atsushi Wakamiya[4], Akinori Saeki[1,3]**

[1] Department of Applied Chemistry, Graduate School of Engineering, The University of Osaka, 2-1 Yamadaoka, Suita, Osaka 565-0871, Japan.

[2] Department of New Energy Science and Engineering, School of Energy and Chemical Engineering, Xiamen University Malaysia, 43900 Sepang, Selangor, Malaysia.

[3] Innovative Catalysis Science Division, Institute for Open and Transdisciplinary Research Initiatives (ICS-OTRI), The University of Osaka, 1-1 Yamadaoka, Suita, Osaka 565-0871, Japan.

[4] Institute for Chemical Research, Kyoto University, Uji, Kyoto 611-0011, Japan.

AUTHOR INFORMATION

Corresponding Author

* saeki@chem.eng.osaka-u.ac.jp (A.S.)

This supporting information presents the following contents.

1. Supporting Tables S1–S6 ··· S2

2. Supporting Figures S1–S15 ··· S5

3. Supporting Notes S1–S2 ··· S13

4. Supporting References S1–S4 ··· S14

1. Supporting Tables

Table S1. Summary of viscosity (Pa s) at shear rate of 0.1 s^{-1} of carbon paste with/without SiC and SiC-APTES additive.

Additive [mg]	0	10	20	40	50	60
SiC	24.3	59	152	150	110	181
SiC-APTES	24.3	24.5	23.9	15.1	16.9	34.1

Table S2. Stability data of control C-PSC.[a]

Time [h]	PCE [%]	J_{SC} (mA cm^{-2})	V_{OC} [V]	FF	R_s [Ω cm^2]	R_{sh} [Ω cm^2]	HI
0	14.67	20.33	1.06	0.68	8.85	163.6	0.39
72	14.62	21.75	1.06	0.64	10.13	335.4	0.34
120	14.74	21.79	1.06	0.64	10.59	130.0	0.44
240	14.75	21.52	1.06	0.65	9.86	337.6	0.36
408	13.64	21.81	1.06	0.59	12.01	305.1	0.29
792	14.77	22.83	1.06	0.61	11.52	821.8	0.39
840	14.30	22.93	1.05	0.60	12.81	2368.2	0.46
1008	14.55	22.85	1.07	0.60	12.54	105.7	0.40
1392	11.86	22.64	1.02	0.52	11.12	269.2	0.08
1680	8.61	22.54	1.02	0.37	33.00	125.5	0.24
1704	10.02	22.78	1.04	0.42	23.88	184.8	0.26
1728	8.04	22.13	1.02	0.36	34.87	80.5	0.20

[a] FTO/MeO-2PACz/Al_2O_3/$FA_{0.85}MA_{0.1}Cs_{0.05}PbI_3$/$C_{60}$/Cr/Carbon stored in an ambient condition (20–25 °C and 40–60% RH) without encapsulation.

Table S3. Stability data of SiC-added (30 mg) C-PSC.[a]

Time [h]	PCE [%]	J_{SC} (mA cm^{-2})	V_{OC} [V]	FF	R_s [Ω cm^2]	R_{sh} [Ω cm^2]	HI
0	11.38	21.66	1.05	0.50	16.35	243.0	0.27
24	13.84	22.33	1.04	0.60	9.94	640.6	0.47
96	11.78	23.76	1.06	0.47	14.69	207.6	0.08
192	12.67	23.68	1.07	0.50	12.06	243.2	0.10
360	11.80	21.86	1.07	0.50	15.28	237.9	0.09
912	13.61	24.46	1.07	0.52	12.29	231.7	0.10
1032	14.14	22.10	1.04	0.61	6.33	661.5	0.45
1080	14.38	22.26	1.05	0.62	7.17	636.7	0.39
1104	14.61	22.64	1.05	0.61	6.52	202.8	0.40
1200	14.72	23.19	1.04	0.61	5.67	197.2	0.36
1248	13.46	25.08	1.06	0.51	14.18	234.8	0.34
1344	14.33	22.08	1.05	0.62	6.61	423.9	0.37
1416	13.79	21.59	1.06	0.60	8.06	494.2	0.40
1536	14.06	21.57	1.06	0.62	6.78	523.0	0.39
1608	13.63	21.6	1.05	0.60	6.85	332.0	0.40
1704	13.52	21.38	1.05	0.60	6.60	251.3	0.36
1800	13.97	21.78	1.06	0.61	6.52	597.1	0.40
1872	12.89	21.63	1.05	0.57	9.49	501.9	0.34
1944	13.33	21.47	1.05	0.59	6.88	385.6	0.38
2064	12.49	20.60	1.05	0.58	6.84	523.9	0.34

[a] FTO/MeO-2PACz/Al_2O_3/$FA_{0.85}MA_{0.1}Cs_{0.05}PbI_3$/$C_{60}$/Cr/Carbon stored in an ambient condition (20–25 °C and 40–60% RH) without encapsulation.

Table S4. Stability data of SiC-APTES-added (10 mg) C-PSC.[a]

Time [h]	PCE [%]	J_{SC} (mA cm^{-2})	V_{OC} [V]	FF	R_s [Ω cm^2]	R_{sh} [Ω cm^2]	HI
0	15.09	22.02	1.06	0.65	3.23	221.8	0.29
96	16.84	22.89	1.07	0.69	3.81	337.8	0.28
168	16.44	23.66	1.08	0.65	4.03	358.0	0.31
192	16.70	23.10	1.07	0.67	4.3	272.0	0.36
360	17.81	24.15	1.09	0.68	3.92	212.7	0.36
504	18.01	24.23	1.09	0.68	5.43	342.7	0.29
528	18.45	24.55	1.08	0.69	4.10	253.9	0.41
600	16.85	24.48	1.09	0.63	6.12	105.4	0.28
672	16.90	24.02	1.09	0.65	4.96	332.9	0.22
840	13.16	21.35	1.08	0.57	13.95	229.1	0.30
1056	15.47	23.16	1.08	0.62	4.01	216.5	0.39
1392	16.64	24.02	1.08	0.64	5.63	215.3	0.44
1512	18.12	24.40	1.10	0.68	4.79	1124.3	0.44
1560	15.96	23.47	1.08	0.63	8.24	191.3	0.42
1584	17.04	24.07	1.09	0.65	5.98	249.9	0.40
1680	14.82	24.25	1.06	0.57	9.84	239.7	0.30
1728	13.50	22.58	1.07	0.56	9.74	251.7	0.31
1824	16.85	23.12	1.08	0.67	4.57	349.1	0.40
1896	16.02	22.62	1.09	0.65	5.00	541.9	0.42
1968	15.25	22.53	1.08	0.63	5.45	439.4	0.38
2064	14.77	21.68	1.10	0.62	6.05	223.6	0.45

[a] FTO/MeO-2PACz/Al_2O_3/$FA_{0.85}MA_{0.1}Cs_{0.05}PbI_3$/$C_{60}$/Cr/Carbon stored in an ambient condition (20–25 °C and 40–60% RH) without encapsulation.

Table S5. Summary of SiC-added C-PSC.[a]

Additive [mg]	PCE [%]	J_{SC} [mA cm^{-2}]	V_{OC} [V]	FF	R_s [Ω cm^2]	HI
0	14.75 (12.49±0.98)	21.52 (19.94±0.92)	1.059 (1.059±0.031)	0.646 (0.593±0.032)	9.86 (8.39±1.92)	0.36 (0.40±0.12)
10	14.49 (12.47±1.17)	22.81 (21.19±1.54)	1.041 (1.068±0.024)	0.610 (0.555±0.050)	10.83 (8.15±2.17)	0.31 (0.41±0.10)
20	14.31 (13.25±0.98)	22.65 (20.95±1.21)	1.040 (1.067±0.025)	0.607 (0.593±0.046)	8.84 (7.65±0.97)	0.39 (0.35±0.18)
30	16.05 (14.50±0.98)	22.55 (21.75±0.53)	1.050 (1.057±0.008)	0.677 (0.630±0.032)	6.27 (7.84±1.52)	0.09 (0.14±0.03)
40	9.53 (8.03±1.49)	21.98 (21.72±0.26)	0.950 (0.87±0.080)	0.456 (0.425±0.035)	6.10 (11.34±5.24)	0.18 (0.14±0.03)
50	9.62 (8.15±1.47)	21.78 (20.31±1.47)	0.960 (0.925±0.035)	0.460 (0.430±0.030)	13.72 (8.10±1.61)	0.10 (0.24±0.14)
60	7.00 (5.25±2.13)	17.34 (15.04±5.83)	0.920 (0.937±0.030)	0.438 (0.370±0.051)	16.00 (21.53±4.00)	0.11 (0.08±0.02)

[a] FTO/MeO-2PACz/Al_2O_3/$FA_{0.85}MA_{0.1}Cs_{0.05}PbI_3$/$C_{60}$/Cr/Carbon. The average and standard deviation were calculated for 3–8 devices for each.

Table S6. Summary of SiC-APTES-added C-PSC.[a]

Additive [mg]	PCE [%]	J_{SC} [mA cm^{-2}]	V_{OC} [V]	FF	R_s [Ω cm^2]	HI
0	14.75 (12.49±0.98)	21.52 (19.94±0.92)	1.059 (1.059±0.031)	0.646 (0.593±0.032)	9.86 (8.39±1.92)	0.36 (0.40±0.12)
10	18.43[b] (17.11±0.74)	23.78 (23.65±0.64)	1.061 (1.046±0.010)	0.730 (0.691±0.033)	3.16 (4.84±0.91)	0.04 (0.04±0.01)
20	18.45[b] (17.43±1.16)	24.55 (23.42±1.55)	1.080 (1.077±0.016)	0.696 (0.690±0.012)	4.10 (4.79±0.79)	0.41 (0.27±0.15)
30	18.09 (17.06±0.78)	23.83 (24.44±0.79)	1.060 (1.063±0.005)	0.716 (0.660±0.043)	3.22 (5.44±1.71)	0.07 (0.06±0.01)
40	15.97 (15.35±0.47)	22.41 (23.23±1.12)	1.064 (1.047±0.008)	0.670 (0.630±0.040)	6.50 (7.17±0.81)	0.14 (0.12±0.02)
50	11.38 (9.17±1.96)	21.66 (20.93±0.56)	1.051 (1.002±0.028)	0.500 (0.430±0.075)	16.35 (16.91±2.64)	0.27 (0.12±0.09)
60	5.03 (3.72±1.30)	18.01 (18.43±0.42)	0.840 (0.645±0.195)	0.330 (0.310±0.020)	14.66 (11.13±3.53)	0.05 (0.04±0.01)

[a] FTO/MeO-2PACz/Al_2O_3/$FA_{0.85}MA_{0.1}Cs_{0.05}PbI_3$/$C_{60}$/Cr/Carbon. The average and standard deviation were calculated for 3–8 devices for each. [b] PCE=18.43% obtained for 10 mg rather than 18.45% obtained for 20 mg was collected as the best device in the main text (Table 1), because the latter showed a much higher HI than the former.

2. Supporting Figures

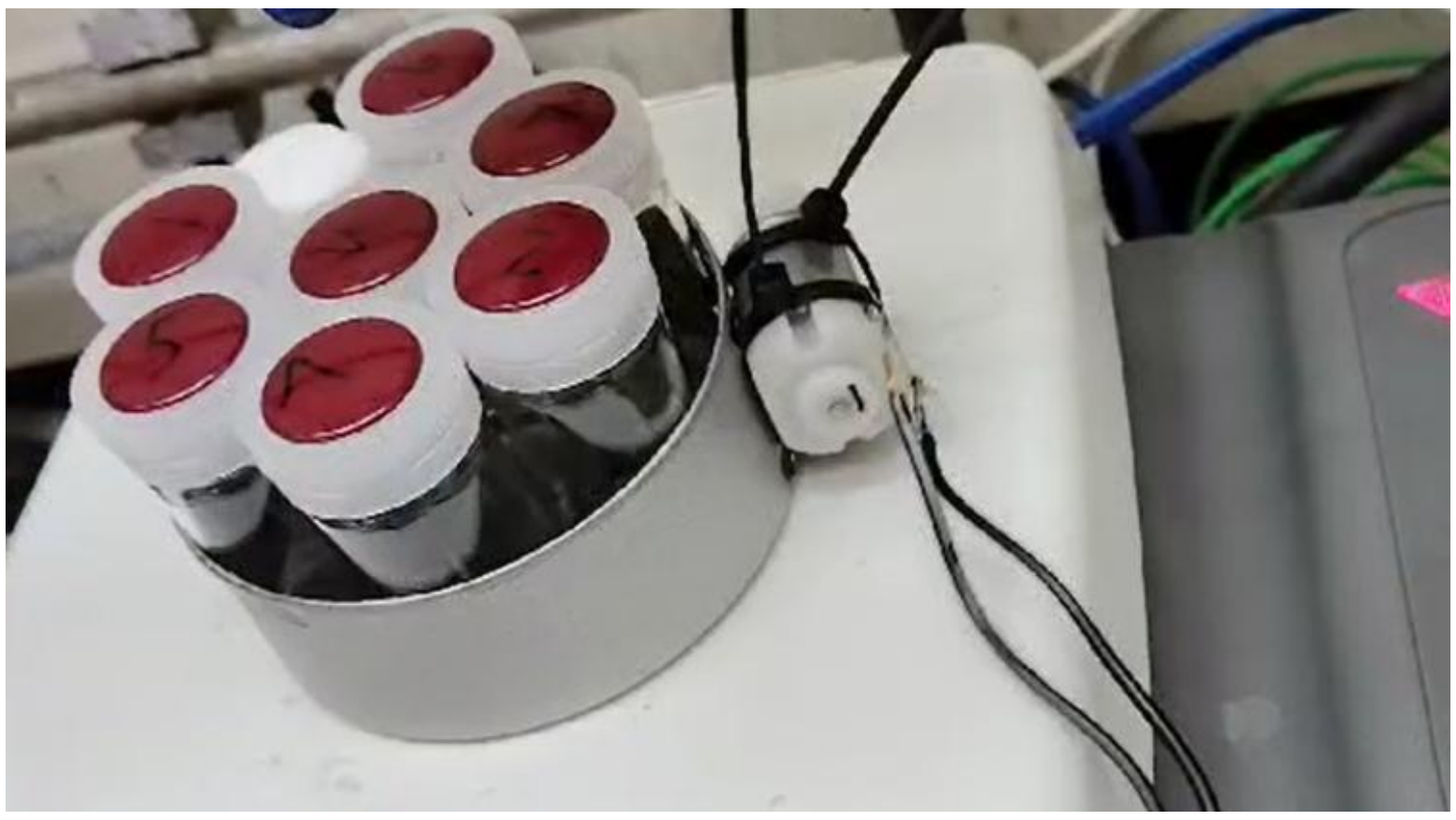

Figure S1. A home-built vibro-heating system used to functionalize SiC. The temperature of the heater is 130 °C.

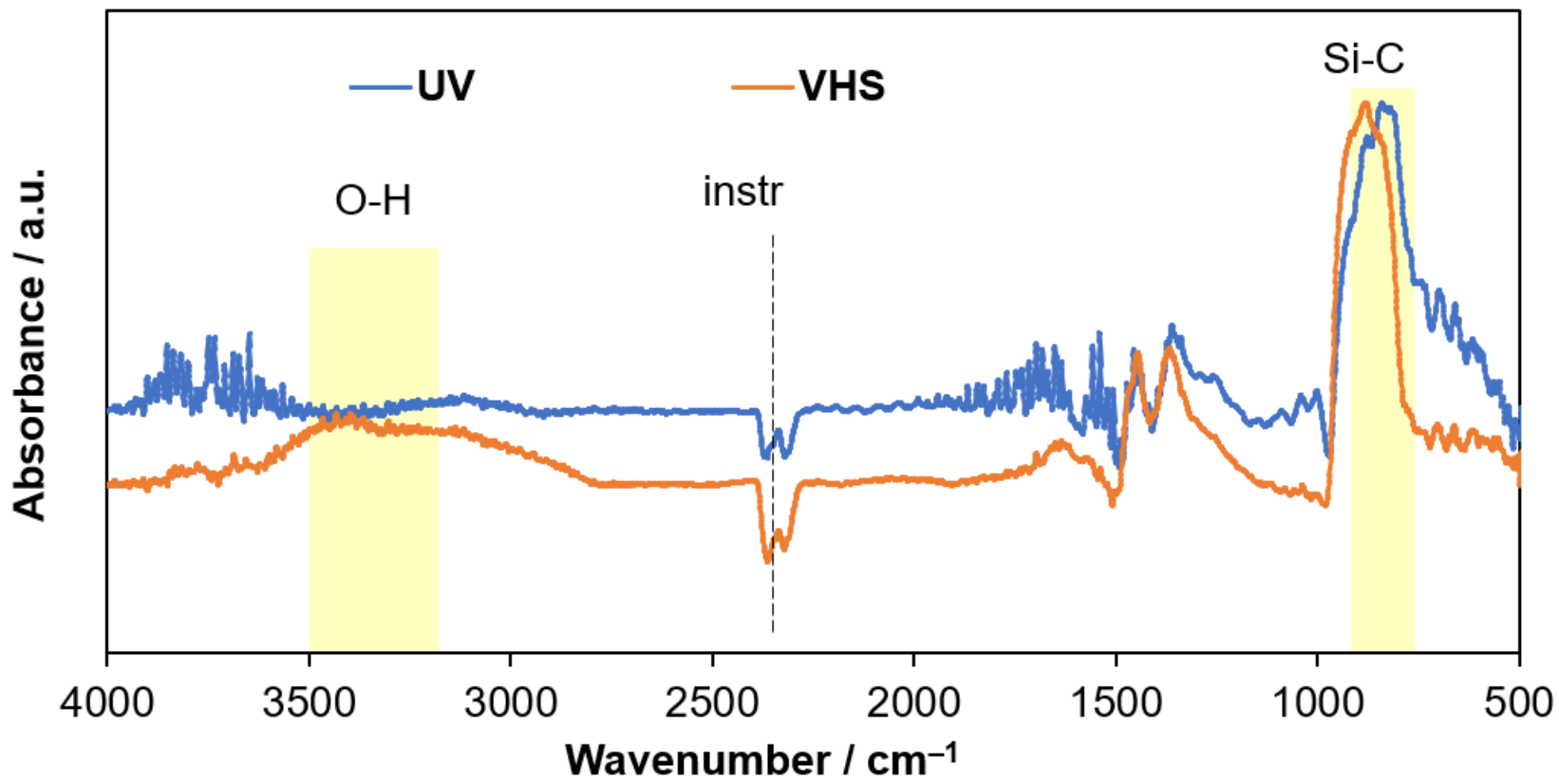

Figure S2. FTIR spectra of SiC-OH powders treated by UV light (UV) and vibro-heating system (VHS).

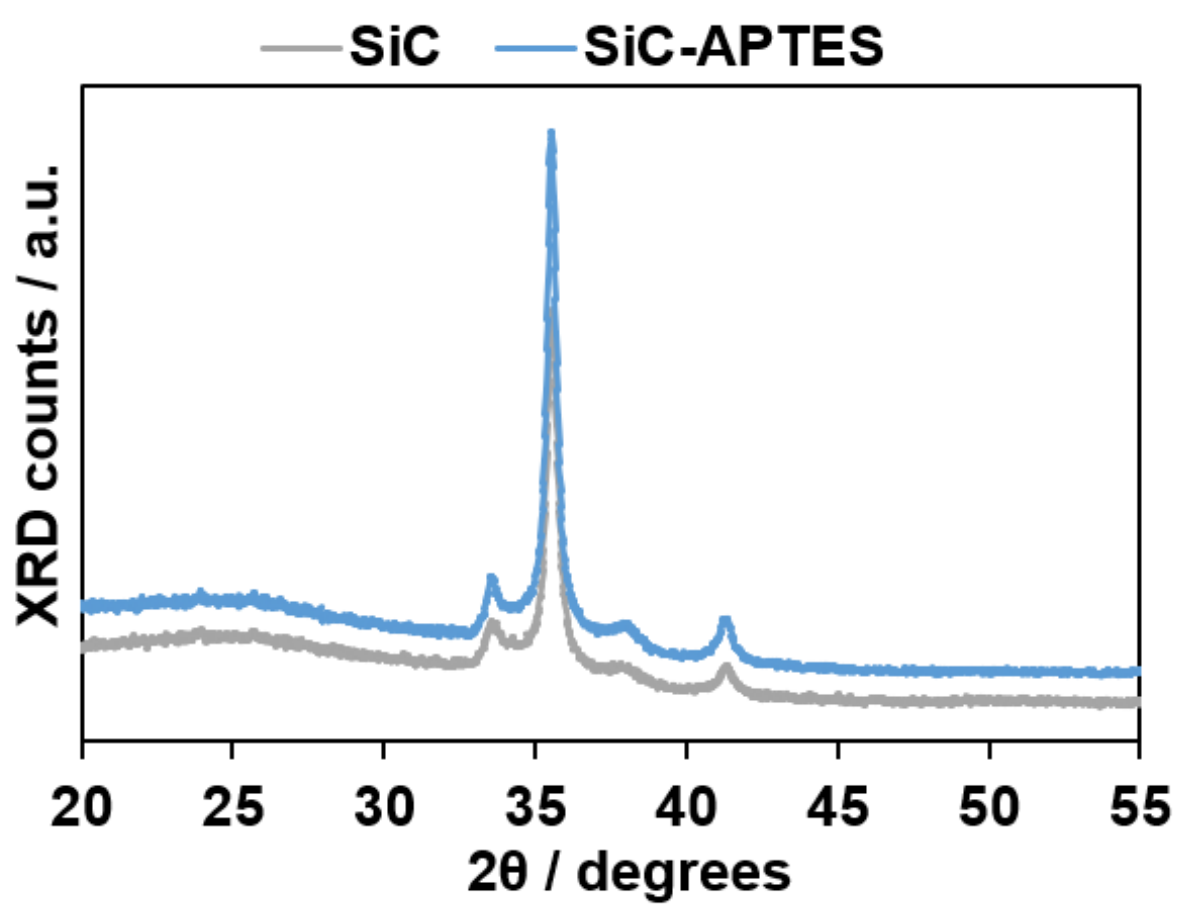

Figure S3. XRD patterns of SiC and SiC-APTES powders.

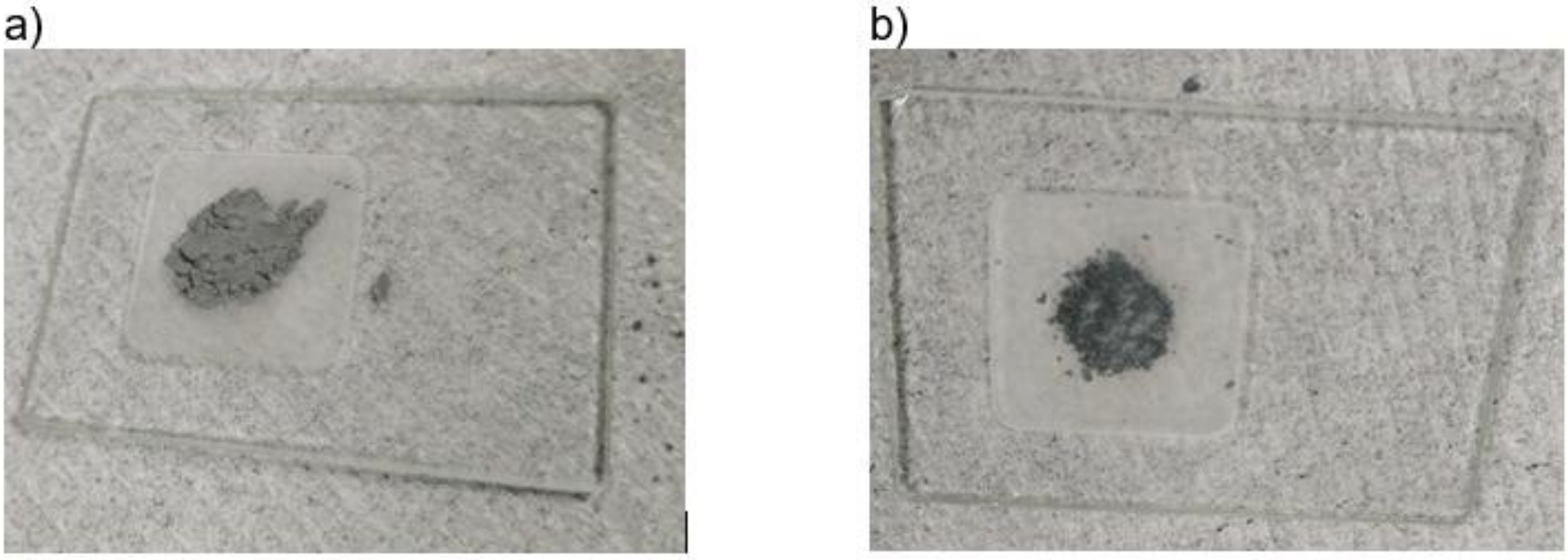

Figure S4. Photographs of (a) SiC and (b) SiC-APTES powders.

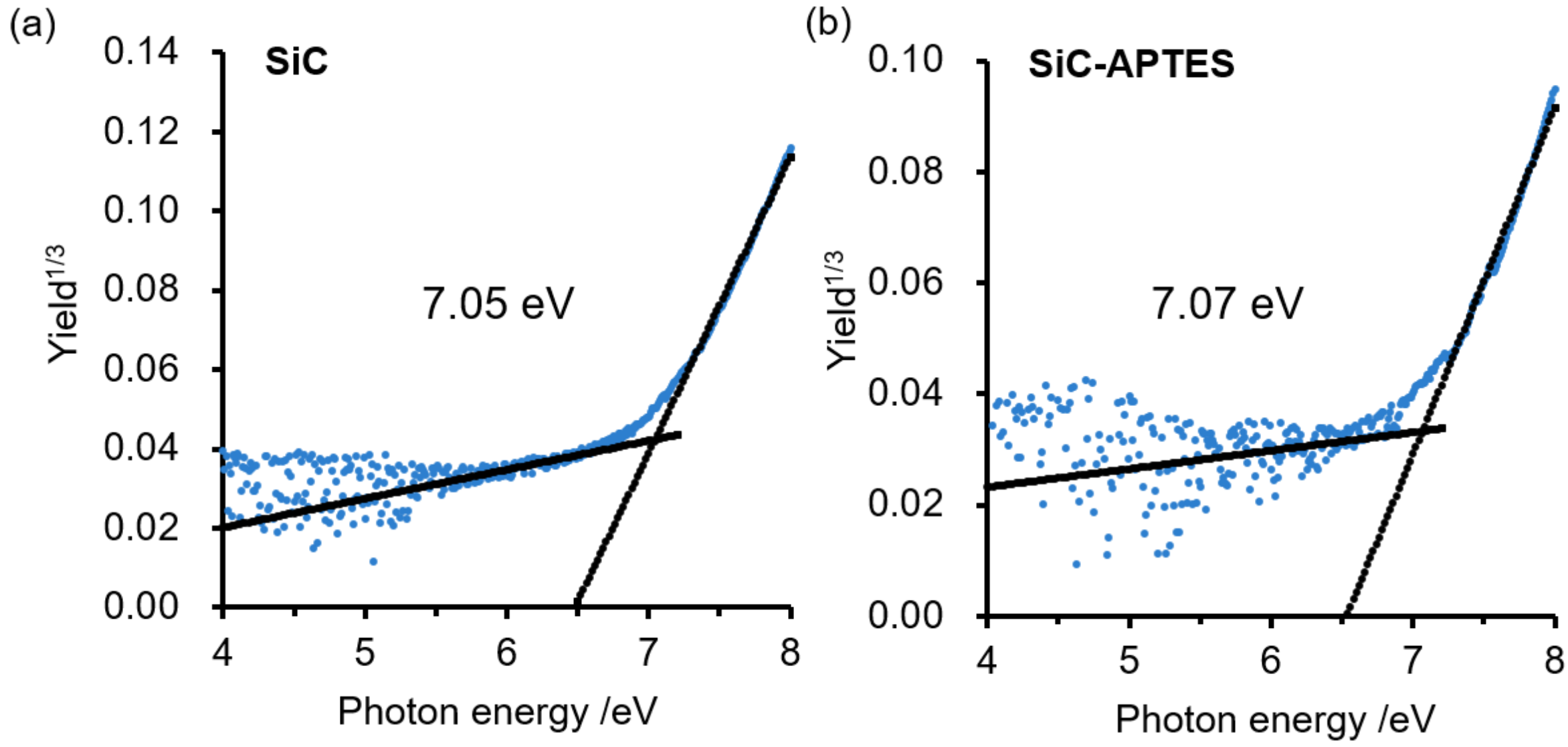

Figure S5. PYS data of SiC and SiC-APTES powder.

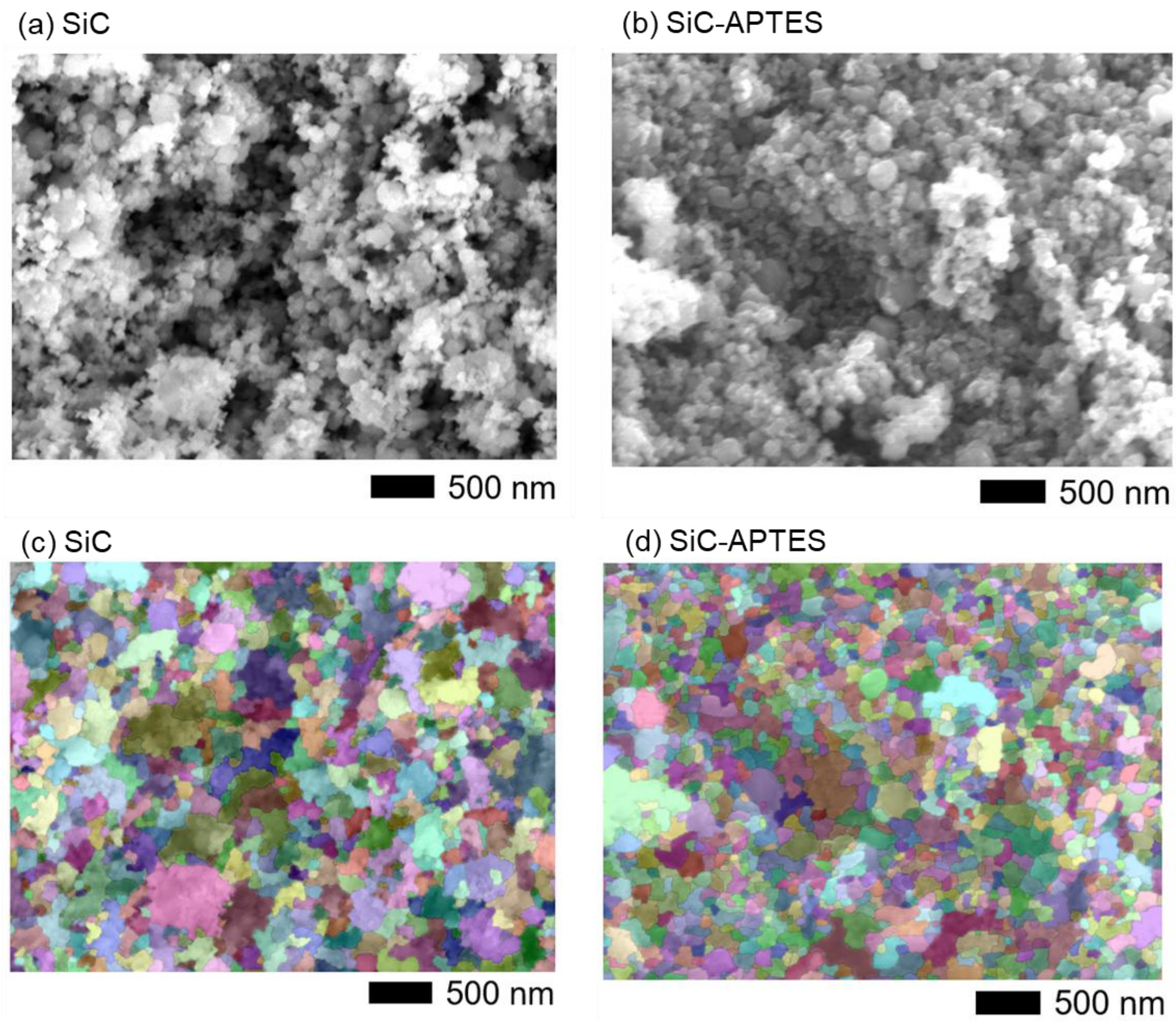

Figure S6. (a)(b) Original and (c)(d) digitized SEM images of SiC and SiC-APTES powders analyzed by ImageJ.

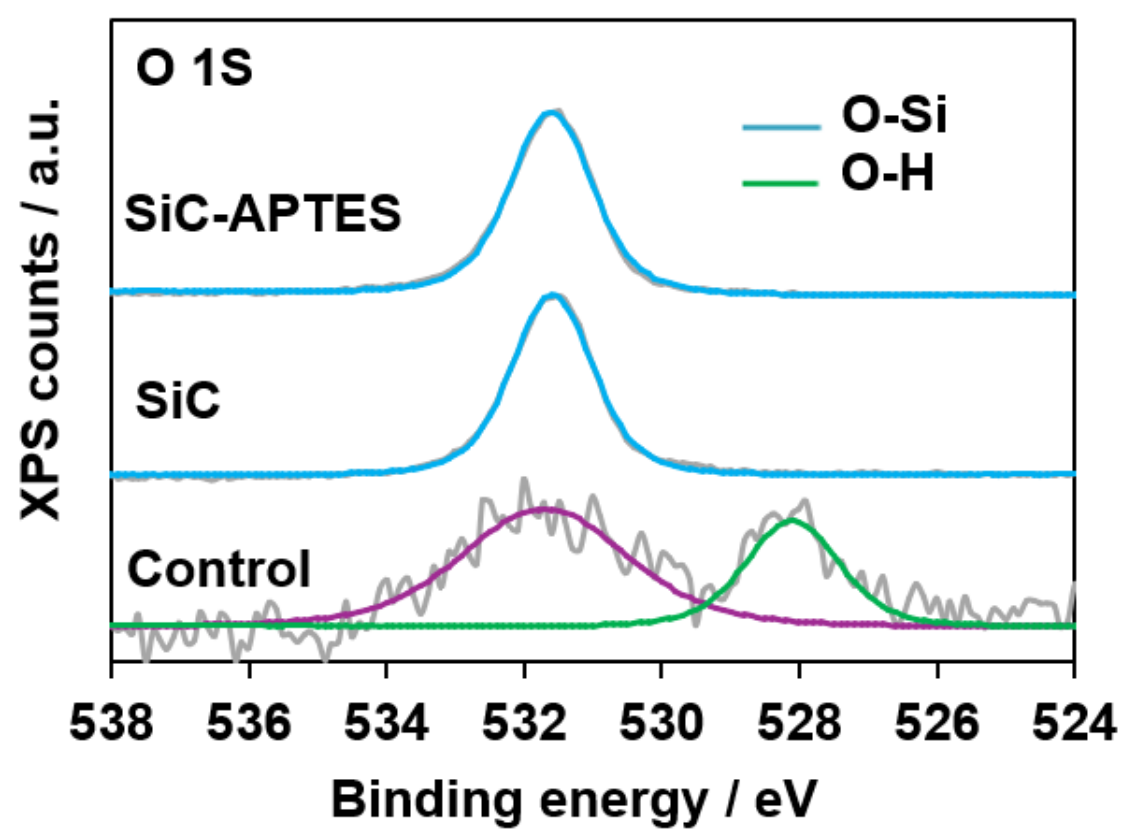

Figure S7. High- resolution XPS scans of (a) O 1s.

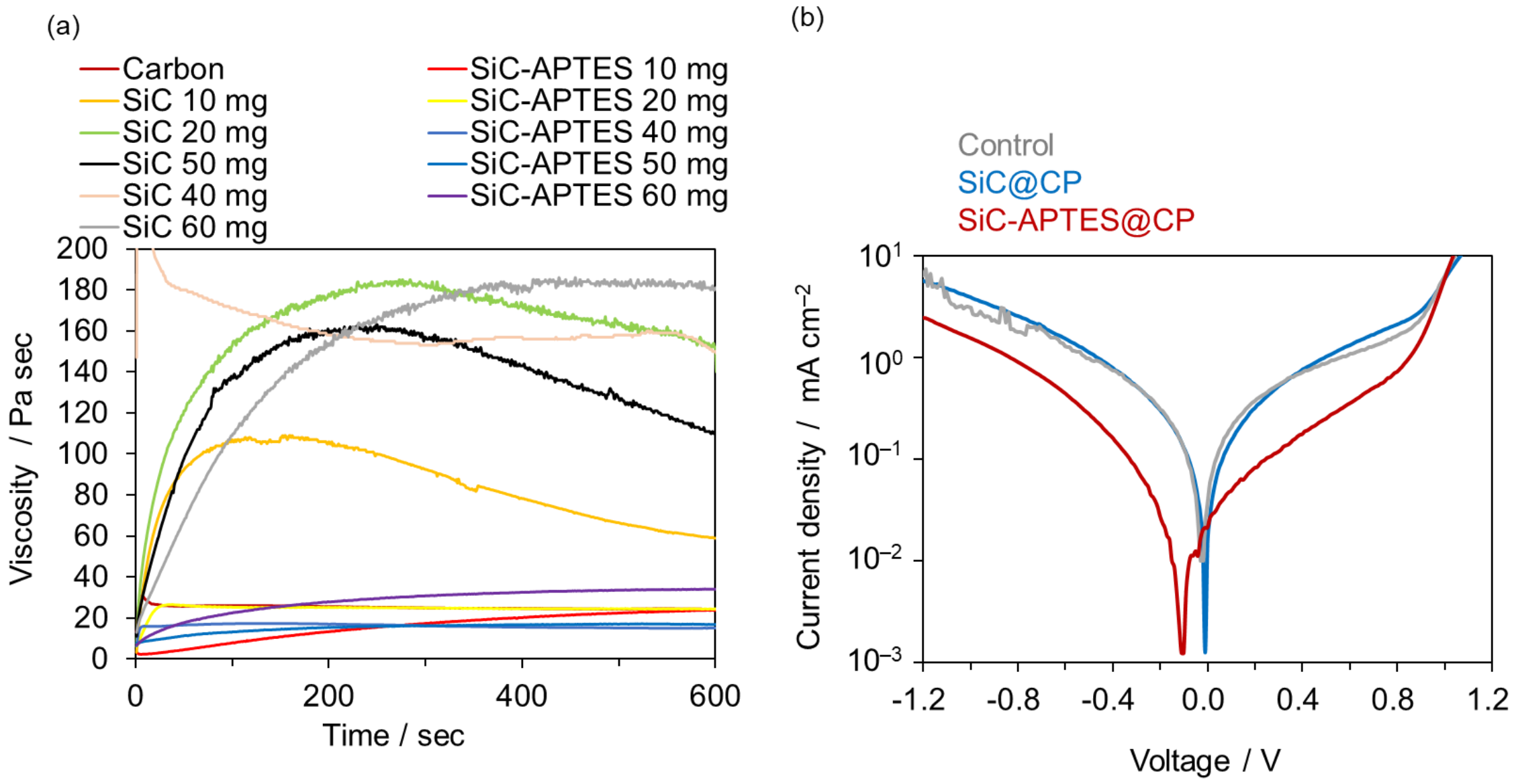

Figure S8. (a) Viscosity of carbon pastes prepared without and with different amounts of SiC or SiC-APTES additives. (b) Dark *JV* curves of PSC devices made with carbon paste (CP) with/without SiC and SiC-APTES.

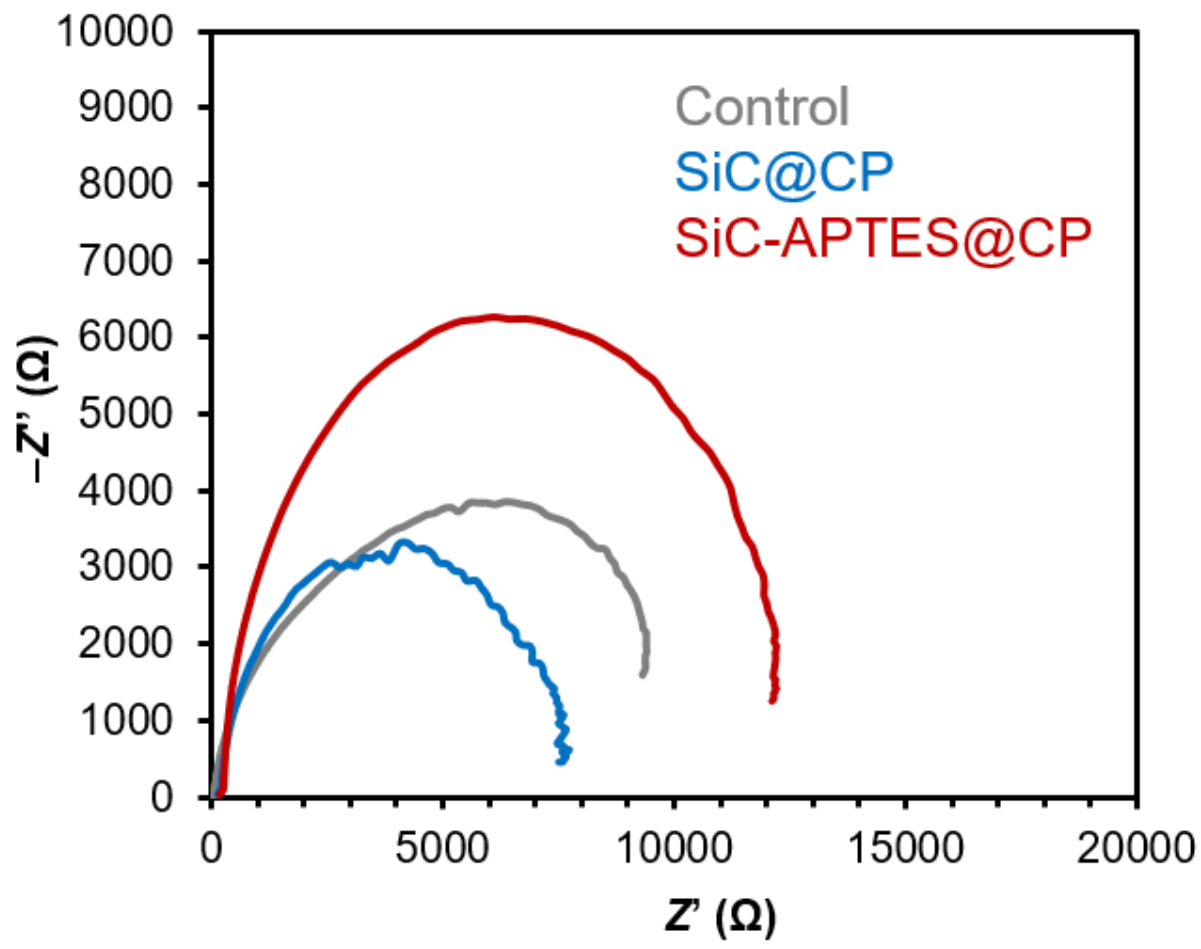

Figure S9. Electrochemical impedance spectroscopy (EIS) measurements of carbon-electrode PSC devices with/without SiC and SiC-APTES. 21 Hz–1 MHz.

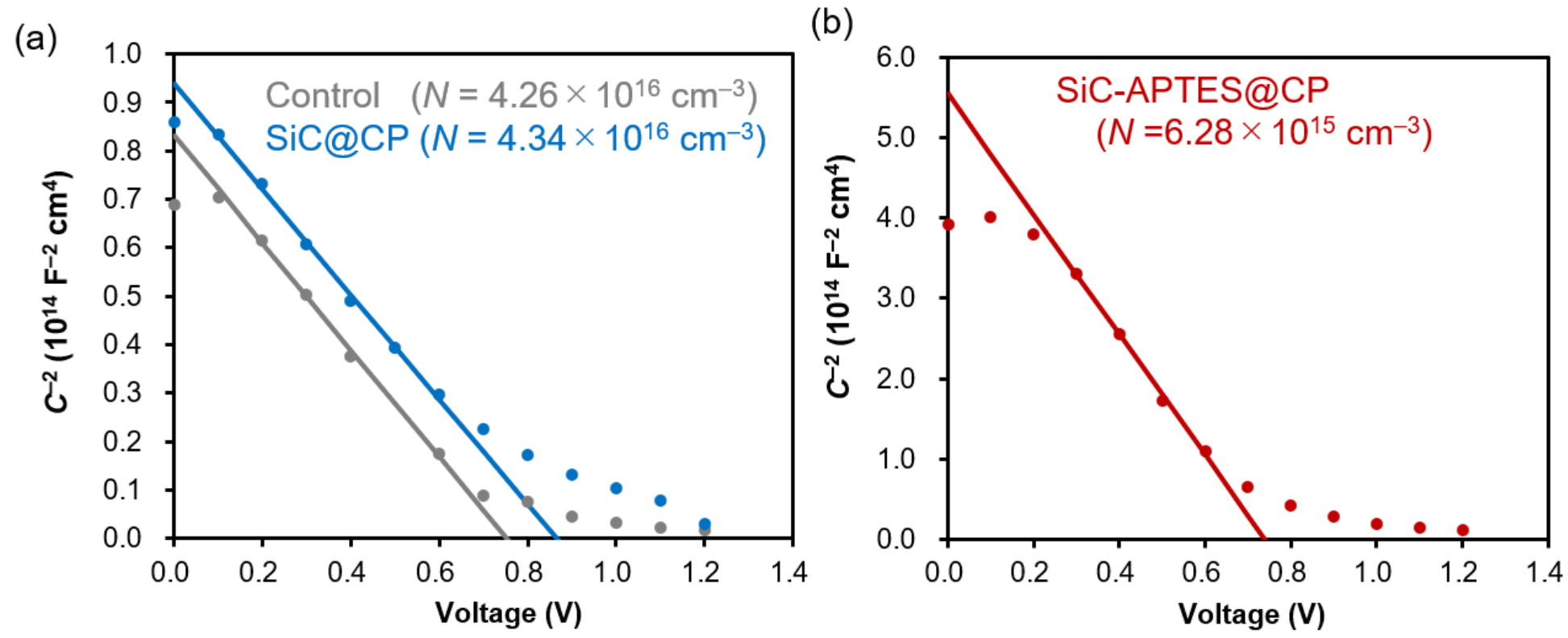

Figure S10. Mott-Schottky analyses of carbon-electrode PSC devices with/without SiC and SiC-APTES. *C*: Capacitance.

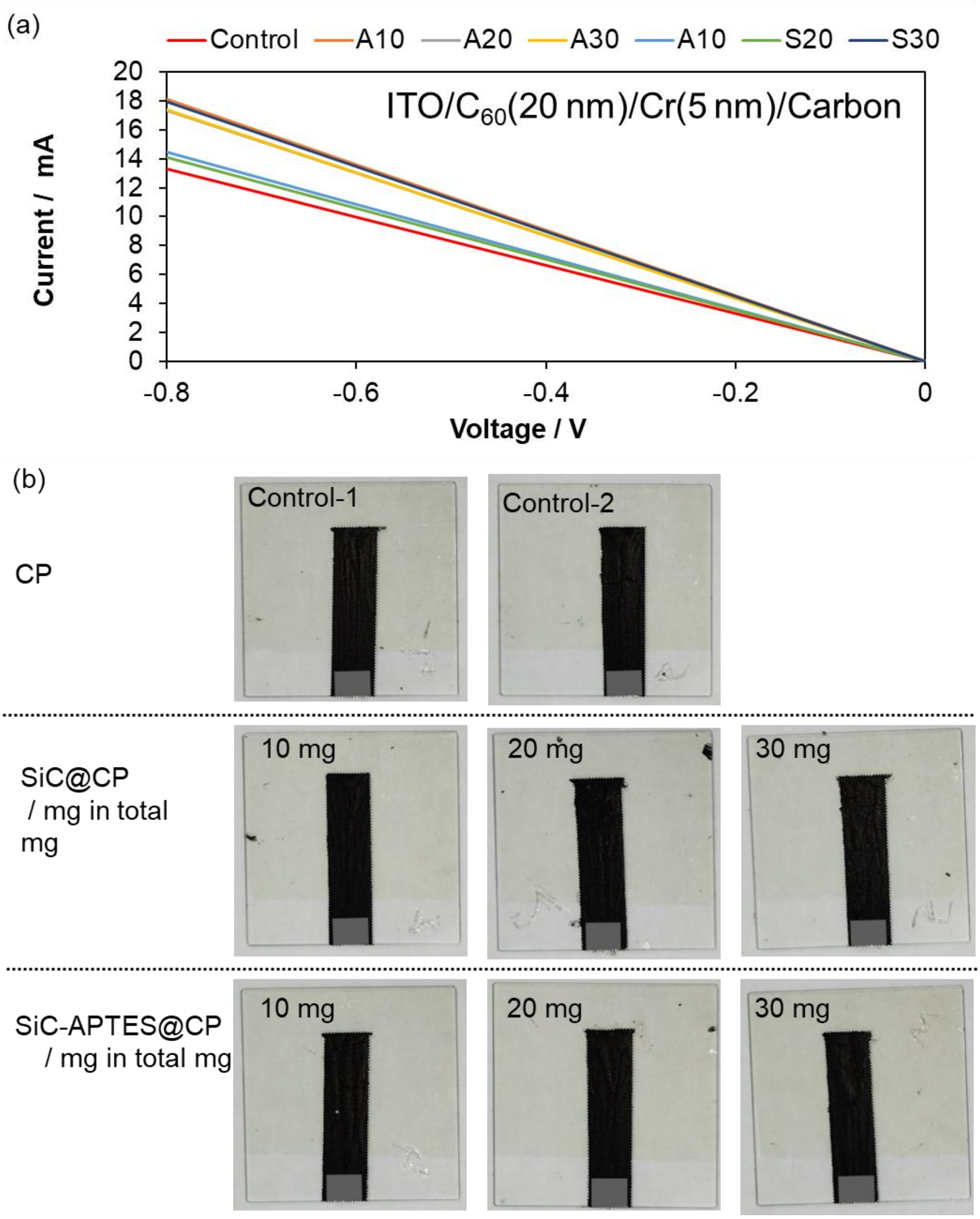

Figure S11. (a) *I-V* curves of the samples with structure: ITO/C_{60} (20 nm)/Cr (5 nm)/Carbon w/wo additives. (b) Pictures of the samples for different amount of SiC or SiC-APTES-loaded carbon electrodes.

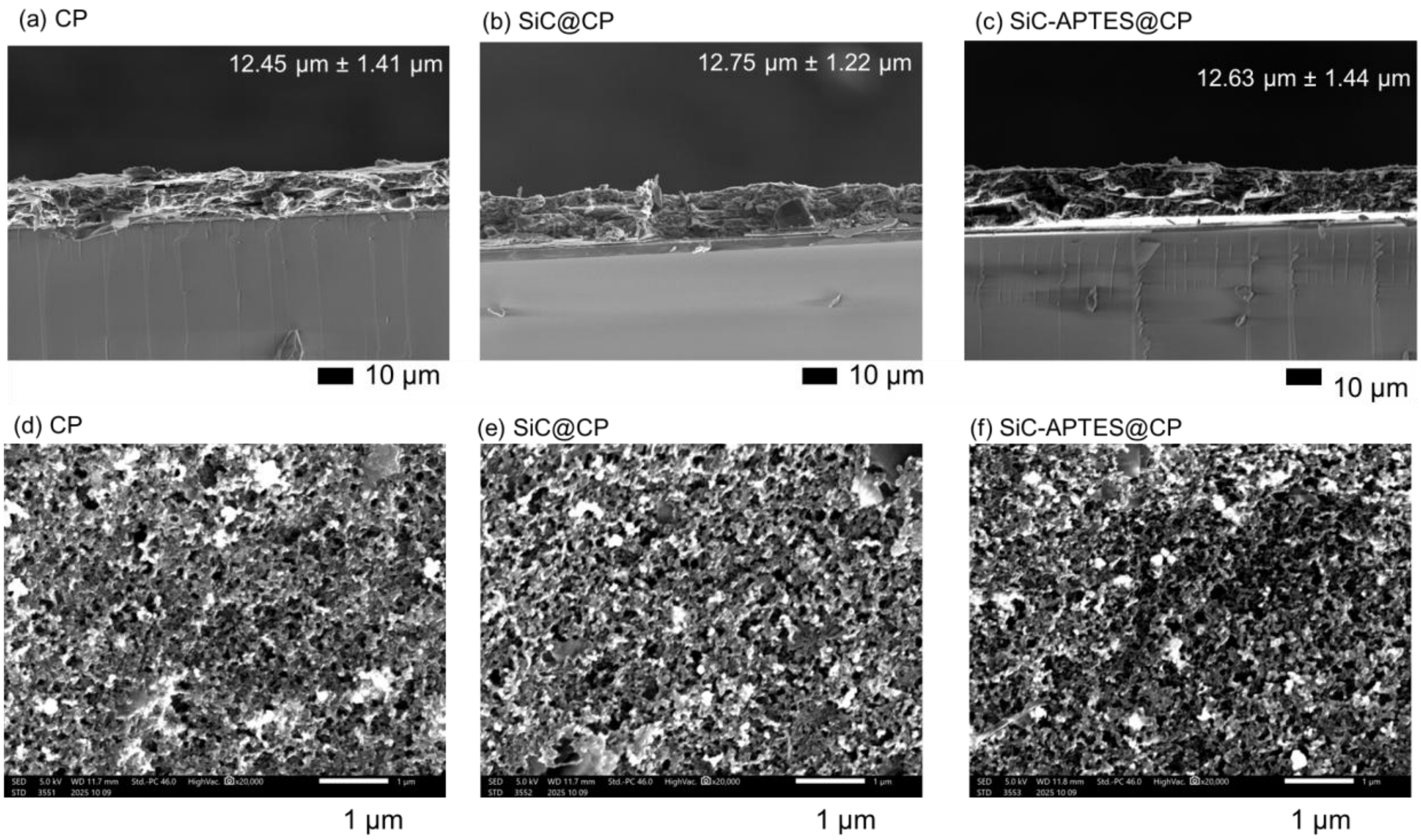

Figure S12. (a)-(c) Cross-sectional and (d)-(f) top view SEM images of PSC devices made with carbon paste with/without SiC and SiC-APTES.

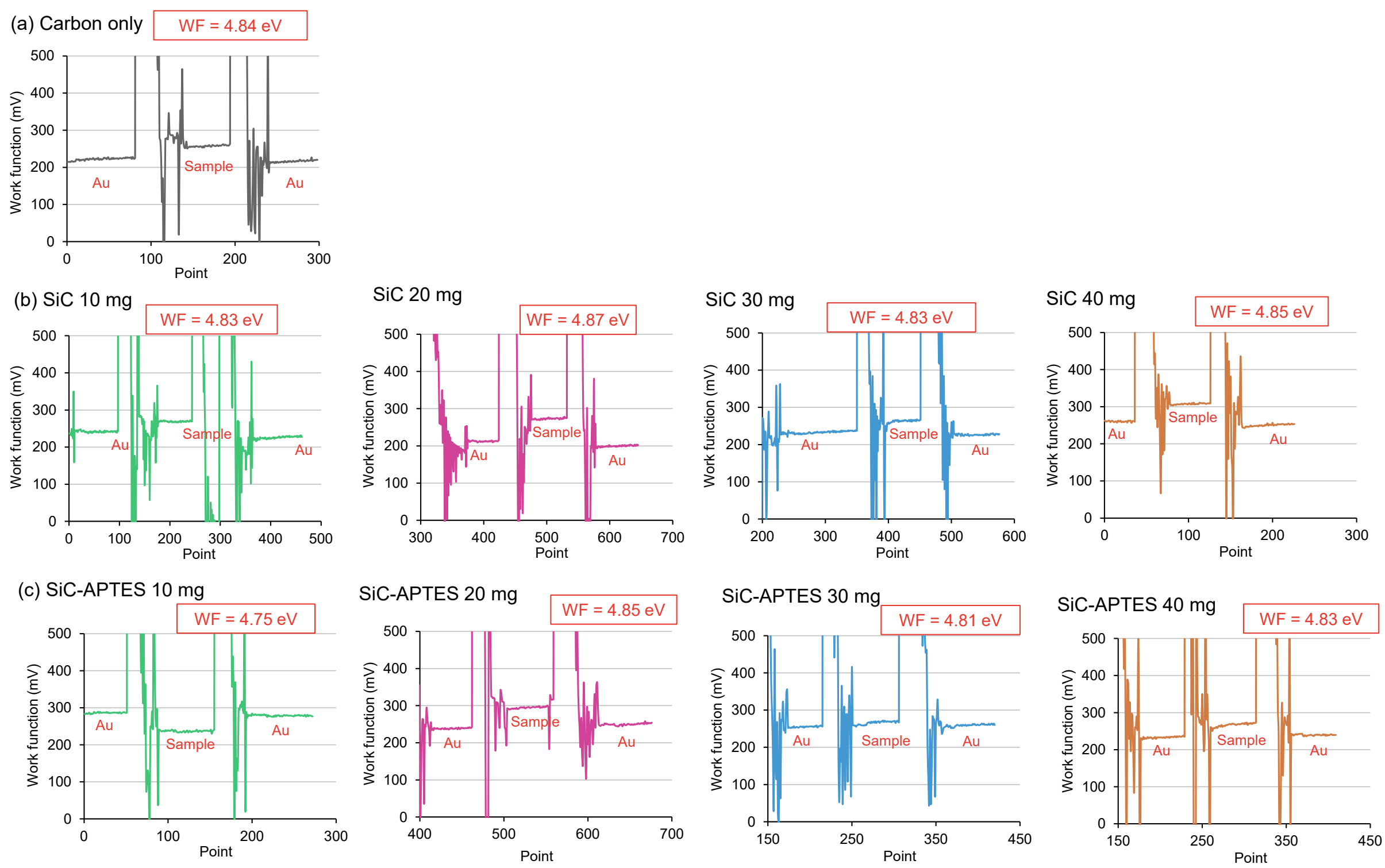

Figure S13. Kelvin probe (KP)measurement data of (a) control carbon electrode, (b) carbon electrode with SiC, and (c) carbon electrode with SiC-APTES. The amount of added SiC or SiC-APTES in total 373 mg carbon composite is given. The Au and sample indicate the reference gold and sample positions, respectively. The work function (WF) is calculated on the basis of WF = 4.80 eV of Au.

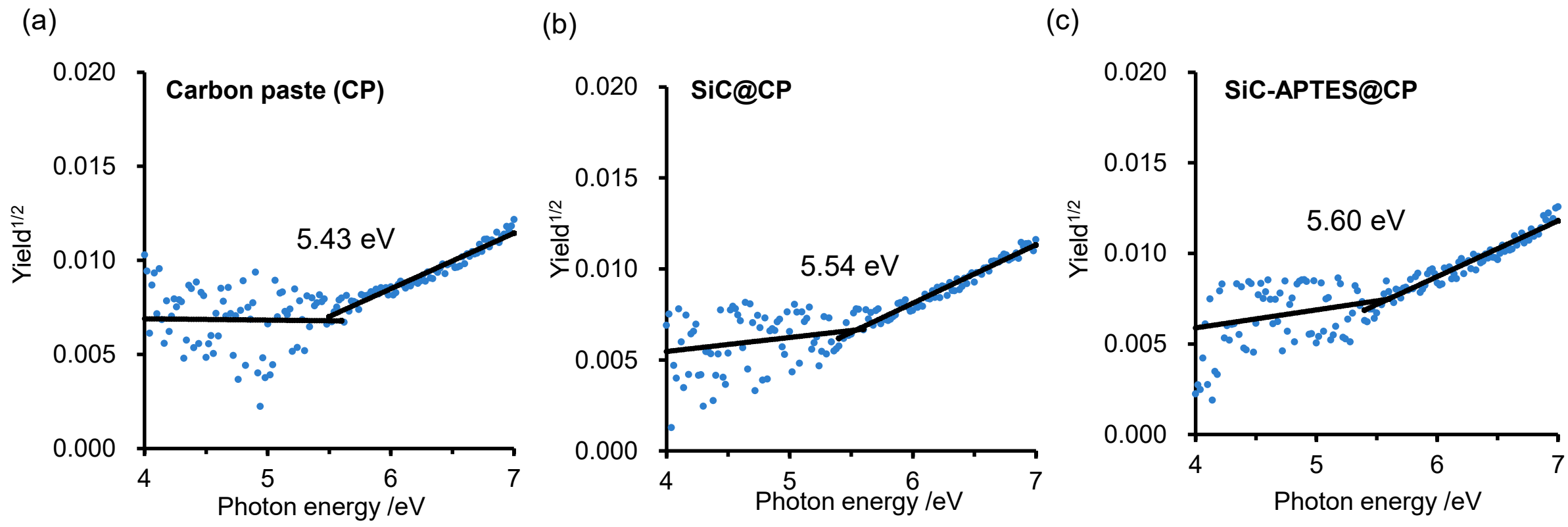

Figure S14. PYS data of (a) carbon paste, (b) carbon paste with SiC, and (c) carbon paste with SiC-APTES. The loaded amount of SiC or SiC-APTES was 30 mg in total 373 mg carbon composite.

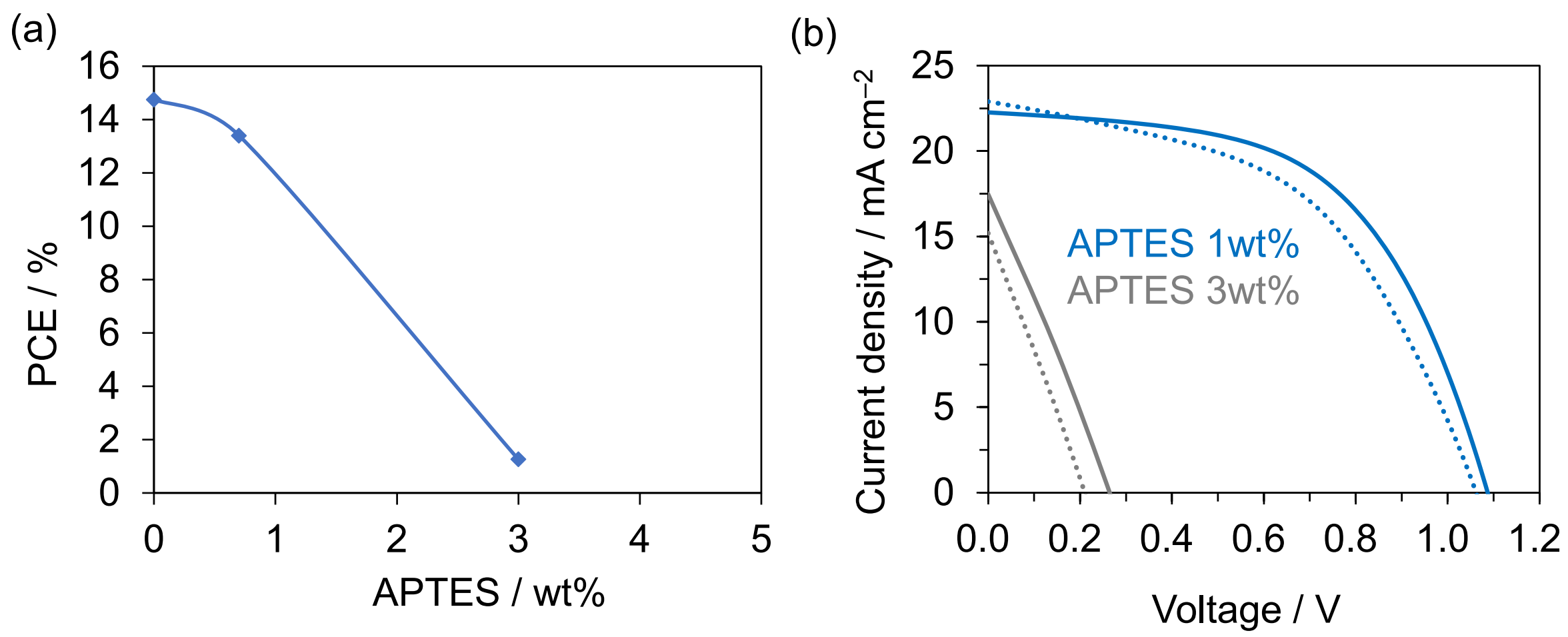

Figure S15. (a) PCE of p-i-n C-PSC with a direct addition of APTES into carbon paste. (b) *J-V* curves of the corresponding devices. The dotted and solid lines are forward and reverse scans, respectively.

3. Supporting Notes

Supporting Note S1: XPS analysis

APTES is (3-aminopropyl)triethoxysilane, $H_2N–CH_2–CH_2–CH_2–Si(OCH_2CH_3)_3$, consisting of a primary amine headgroup linked by a three-carbon propyl chain to a triethoxysilyl anchoring group. The presence of the N 1s XPS peak (**Figure 2a**) after functionalization is an indicator of successful attachment of APTES to SiC[S1–S3]. This is confirmed for the carbon paste with addition of SiC-APTES only additionally, both samples with addition of SiC contain Si-C and Si-C-O groups on the Si 2p scan (**Figure 2b**), while these are absent for the control carbon paste sample. **Figure S7** shows high- resolution XPS O 1s scans of control carbon paste deposited on FTO and with SiC and SiC-APTES additives respectively. The O 1s scans of all three samples contain O-Si peaks. The plain carbon paste contains exposed hydroxyl groups from residual cellulose binder, partially oxidized carbon black surfaces, or adsorbed atmospheric moisture. In contrast, when SiC or SiC-APTES is added, these native -OH groups are largely replaced or covered by the silane-derived Si-O-C and Si-O-Si networks. Presumably, those O-Si peaks are hidden under the very strong O–C peak and are practically indistinguishable from the oxygen in the carbon paste binder itself. As for the C scan (**Figure 2c**), we identify additional peaks in SiC- containing samples as C-Si bonds and SiO_xC_y, and for the control sample additional C-OH peak[S2](in accordance with O 1s scan), but those peaks also can be attributed to adventitious carbon[S4]. Regardless of the explanation, it is irrelevant to this work as the goal of XPS was to confirm the presence of SiC-APTES in the carbon pastes after preparation that was shown with Si 2p and N 1S scans.

Supporting Note S2: Conductivity analysis (Figure 5c)

The ITO/glass substrates (overall size 1.6 × 1.6 cm^2) were etched with HCl and Zn along the edges, leaving a central conductive ITO area of 1.1 × 1.6 cm^2.ITO/glass substrate . After evaporation of 20 nm of C_{60} and 5 nm of Cr, carbon pastes (thickness ~ 12 μm) were deposited on the samples with a stripe 0.4×1.5 cm^2. The *I-V* curves were recorded to find conductance (*G*). Area of the carbon electrode is crucial for precise determination of the conductance in this measurement. In order to normalize the areas, Photoshop was used to get the number of pixels that is proportional to the area, then the values were normalized and the correction coefficients (0.95-0.99) were considered in the calculation, and thus, the values in **Figure 5c** are normalized conductivity. The photographs are shown in **Figure S10**.

4. Supporting References

[S1] A. L. S. and K. U. R Yakimova, R M Petoral Jr, G R Yazdi, C Vahlberg, *J. Phys. D. Appl. Phys.* **2007**, *40*, 6435. https://doi.org/10.1088/0022-3727/40/20/S20

[S2] Y. Tak, W. S. Hwang, J. U. Jeong, *J. Korean Ceram. Soc.* **2020**, *57*, 200. https://doi.org/10.1007/s43207-020-00017-0

[S3] Y. Xu, Y. Xin, T. Shirai, *J. Ceram. Soc. Japan* **2022**, *130*, 390. https://doi.org/10.2109/jcersj2.22011

[S4] G. Greczynski, L. Hultman, *Vacuum* **2022**, *205*, 111463. https://doi.org/10.1016/j.vacuum.2022.111463
